# The RNF8/OPTN/KDM6A axis controls macrophage polarization to maintain testicular microenvironment homeostasis

**DOI:** 10.1038/s41420-025-02641-3

**Published:** 2025-07-24

**Authors:** Yanan Guo, Peng Xia, Yixiao Tian, Daosen Fu, Xiaohui Hu, Kun Xie, Wenhao Dong, Wei Zhang, Disheng Liu, Rong Shen, Degui Wang

**Affiliations:** 1 School of Basic Medical Sciences, Lanzhou University, Lanzhou, Gansu China; 2 Reproductive Medicine Center, The First Hospital of Lanzhou University, Lanzhou University, Lanzhou, Gansu China; 3 Department of Urology, The First Hospital of Lanzhou University, Lanzhou University, Lanzhou, Gansu China

**Keywords:** Inflammatory diseases, Ubiquitylation

## Abstract

Dysregulated immune responses may erroneously target normal reproductive tissues, thereby compromising the proper functioning of the reproductive system. Macrophages are the most abundant immune cells in the testes, however, the role of macrophages in spermatogenic function is not yet clear. This study indicated that the increase of pro-inflammatory macrophages impaired the development of spermatogenic cells, and the deficiency of RNF8 led to a proinflammatory state in the testicular microenvironment and diminished sperm production in mice. RNF8 mainly assembled K63-branched ubiquitin chains on autophagy receptor OPTN at K448 thus causing OPTN activation. The increased ubiquitination of OPTN promoted degradation of KDM6A via the autophagy-lysosome pathway, thereby inhibiting macrophage polarization towards the pro-inflammatory type and maintaining an immune privilege state in the testicular microenvironment. This homeostasis could be collapsed once the RNF8-OPTN-KDM6A axis was abnormal, subsequently resulting in remodeling of the testicular microenvironment. This study reveals the underlying mechanism of RNF8 on male reproduction, and the pro-inflammatory microenvironment resulting from RNF8 deficiency hindered spermatogenic cell differentiation, thereby impairing spermatogenic function.

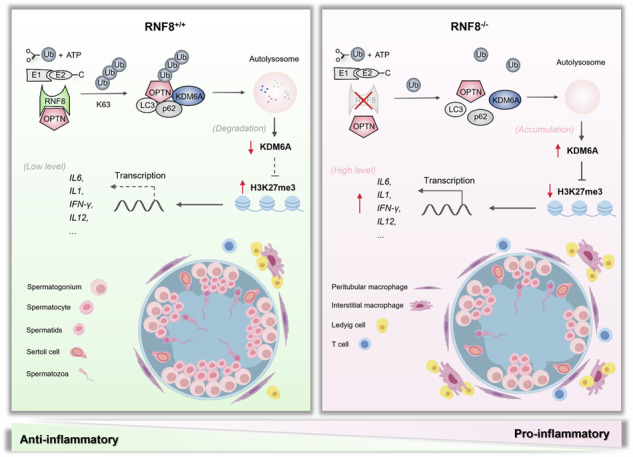

## Introduction

Infertility is a major health problem worldwide. According to the WHO report, about 17.5% of adults (about one-sixth of the global population) are suffering from infertility [1]. Among the etiology of infertility, the male factor is the primary contributing factor for ~50% of couples [2]. Infertility problem has become a world problem.

Our study and Lu et al. found that RNF8 deficiency resulted in male infertility in mice [3, 4], in which we speculated that loss of RNF8 led to defects in histone ubiquitination modification, thus hindering the replacement process of histone into transition proteins and protamine, and induced in abnormal sperm. But soon Hironori Abe et al. proposed that RNF8 was not involved in histone-to-protamine exchange [5]. At present, the impact and mechanisms of RNF8 deficiency on spermatogenic function in mice remain unclear and disputable. We found the increase in macrophages, activation of inflammatory responses, and impaired spermatogenic development in the testes of RNF8-deficient mice via scRNA-seq.

Testicle is a special immune-privileged organ that forms a relatively immunosuppressive microenvironment [6, 7] to protect sperm from being incorrectly recognized and attacked by the immune system. The incidence of asymptomatic inflammation is significantly increased in testicular biopsy of infertile men. In patients with chronic orchitis, there is infiltration of lymphocytes around the seminiferous tubules, damage to tubules, increased levels of autoantibodies and pro-inflammatory mediators, and the presence of polymorphonuclear granulocytes and macrophages in semen [8, 9]. These indicate that the high inflammatory response mediated by immune cells leads to the disruption of immune homeostasis in the testicular microenvironment, ultimately resulting in reduced fertility. The testicular microenvironment is complex and diverse. According to the increasing proportion of asymptomatic inflammation in clinical oligospermia patients and the particularity of the testicles, we mainly conduct research on macrophages, which are the most abundant immune cells in the testicles.

Macrophages are the most abundant immune cells in the testis, which play a key role in regulating local immune response, maintaining tissue homeostasis, and remodeling. Macrophages have multiple intermediate states or activated forms under special conditions in different organs or disease states and can exhibit complex and diverse phenotypic and functional characteristics. Classical M1 and M2 macrophages have been extensively studied [10, 11]. Macrophages in the testis also have the functions of regulating testicular development, steroid production, spermatogenesis, and retinoic acid synthesis [12, 13]. Based on scRNA-seq and spatial transcriptome analysis, some studies suggested that testicular macrophages had different phenotypes from peripheral macrophages. However, the mechanisms by which macrophages regulate spermatogenesis are still limited. Our previous study found that the proportion of pro-inflammatory macrophages in the testes of patients with idiopathic Non-Obstructive Azoospermia (iNOA) was significantly increased and negatively correlated with spermatogenic function [14]. Consequently, we hypothesized that the polarization of testicular macrophages might influence the differentiation of spermatogenic cells.

In this study, we observed an increase in pro-inflammatory macrophages within the testes of RNF8^−/−^ mice, and we noted that the pro-inflammatory macrophages were particularly elevated in patients with oligospermia, prompting us to investigate their role in male infertility. We demonstrated that RNF8 mediated the degradation of KDM6A via the autophagic lysosomal pathway by regulating OPTN activity. KDM6A could influence the epigenetic modification of macrophages and sustain their polarization through modulation of H3K27me3 levels. Loss of RNF8 resulted in increased accumulation of KDM6A, leading to decreased levels of H3K27me3, enhanced transcription of pro-inflammatory factors, and subsequently promoted pro-inflammatory polarization of macrophages. More broadly, pro-inflammatory macrophages reshaped the testicular microenvironment, leading to impaired spermatogenic function and male sterility, as demonstrated in both mouse and human samples.

## Results

### RNF8 deficiency leads to testicular microenvironment disorder and infertility in mice

**Fig. 1:**
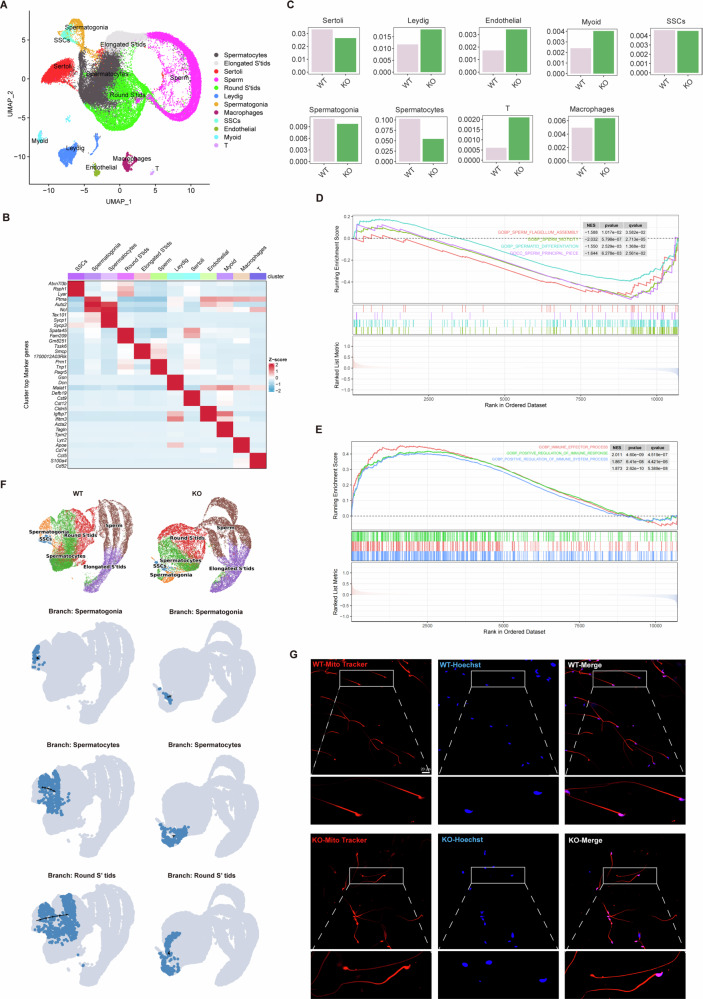
Inhibition of the spermatogenesis process in the microenvironment of RNF8^−/−^ mice testes. **A** Single-cell UMAP clustering diagram of mice testicular tissue. **B** Heat map of differentially expressed genes among different cell types. **C** Analysis of the proportion of different cell types in testicular tissue. **D** GSEA enrichment analysis showed that pathways related to spermatogenic function in KO mice were inhibited. **E** GSEA analysis showed that immune inflammatory response-related pathways were activated in KO mice. **F** Palantir pseudotime developmental trajectory of germ cells in WT and KO mice. **G** Mitochondrial staining of sperm of WT and KO mice, scale bar 20 µm.

To further clarify the exact mechanism of RNF8 gene deletion leading to male infertility, we conducted scRNA-seq analysis on the testicular tissues of RNF8^+/+^ (WT) and RNF8^−/−^ (KO) mice to explore the effects of RNF8 deficiency on mice testis. UMAP dimensionality reduction analysis revealed the distribution of different cell types in the testis (Fig. 1A), and compared the gene expression levels of different cells (Fig. 1B). Cell proportion analysis showed that the proportion of spermatogenic cells was decreased in KO mice, while the proportion of interstitial cells, such as T cells, macrophages, myoid cells, and endothelial cells were increased (Fig. 1C). GSEA analysis performed dysfunction of spermatogenic cells (Fig. 1D) and enhanced immune inflammatory response in KO mice (Fig. 1E).

Pseudotime analysis of germ cells of WT and KO mice was carried out by Palantir algorithm. Cell trajectory distribution showed that there was a significant difference in the process of germ cell differentiation and maturation between the two groups, and sperm differentiation process of KO mice was mainly blocked in the spermatocyte stage (Fig. 1F). In addition, the gene expression of germ cell markers (*Zbtb16*, *Stra8*, *Scp3*, *Acvr1*, *Tssk6*, *Prm1*) in different stages of KO mice was decreased (Supplementary Fig. 1A, B). HE staining showed that the spermatocytes of KO mice were significantly less than those of WT mice (Supplementary Fig. 1C–E), which indicated that the differentiation process of testicular germ cells in KO mice was hindered. Mitochondrial staining and sperm counts were used to observe the number of sperm, results showed that compared with WT, the number of sperm in KO mice was decreased (Fig. 1G, Supplementary Fig. 1F). These results suggest that RNF8 plays a crucial role in spermatogenesis, and its deletion may lead to the differentiation disorder of male spermatogenic cells and the decrease of spermatogenic function.

### Clinical spermatogenesis disorders are associated with elevated levels of inflammation

**Fig. 2:**
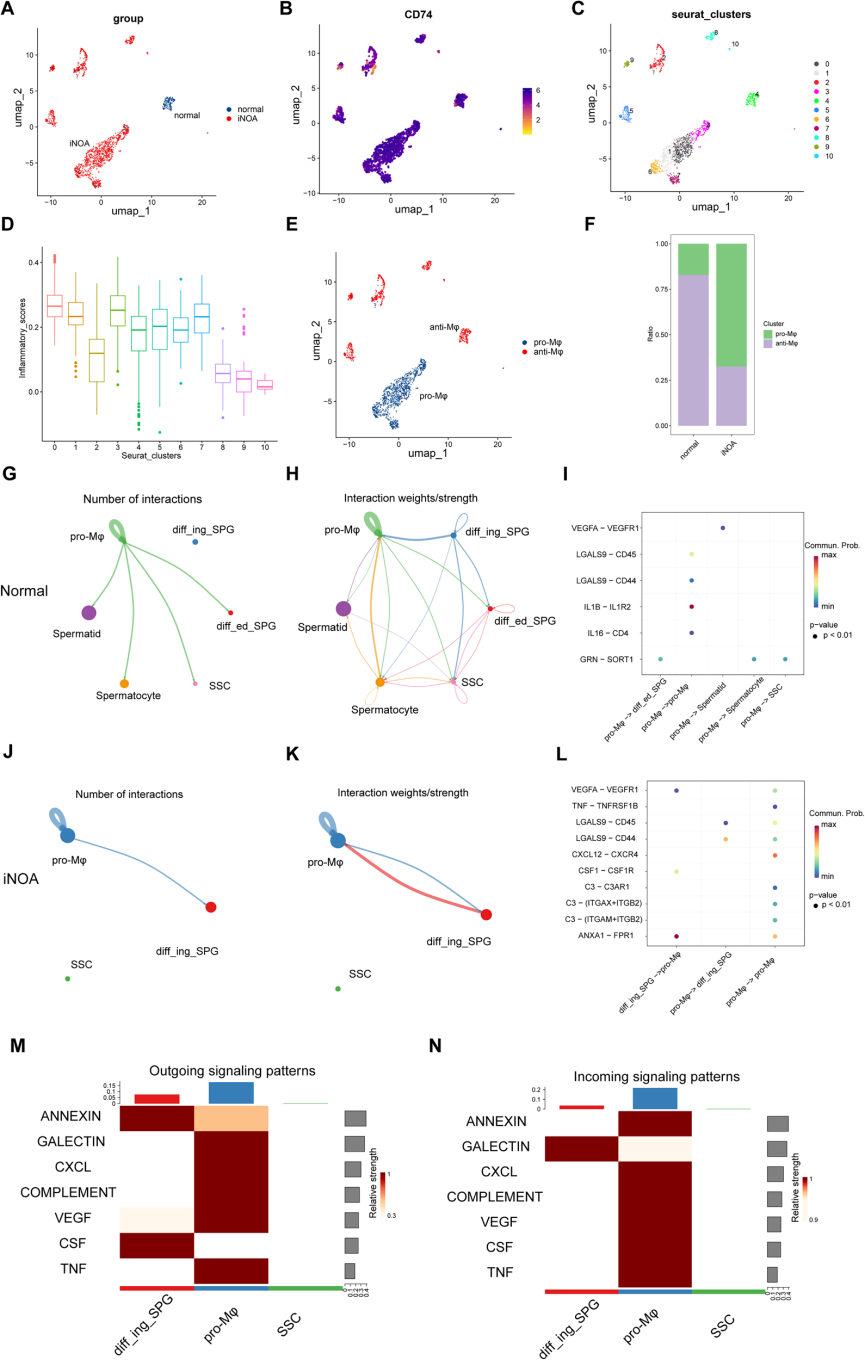
Analysis of cellular communication and macrophage phenotype in the testicular microenvironment of iNOA patients. **A** UMAP clustering diagram of macrophage distribution in testicular tissue of iNOA patients and control group. **B** Analysis of CD74 gene expression in testicular macrophages. **C** Based on the UMAP map of Seurat reclustering, a total of 11 cell groups were subdivided. **D** Based on the inflammation gene set score in MsigDB, the horizontal axis represents cell clustering, and the vertical axis represents the inflammation score value. **E** The distribution of macrophage subtypes (pro-Mφ and anti-Mφ) in the testicular tissue of the control group and iNOA group. **F** The proportion of pro-Mφ and anti-Mφ in the whole macrophage in the testis of the control group and iNOA group. The analysis of intercellular communication in the control group, including communication between pro-Mφ and different spermatogenic cells (**G**), mutual communication between cell groups (**H**), and communication relationship between pro-Mφ and ligand-receptor of spermatogenic cells (**I**). The analysis of intercellular communication in the iNOA group includes the communication between pro-Mφ and different spermatogenic cells (**J**), the mutual communication between cell groups (**K**), and the communication relationship between pro-Mφ and ligand-receptor of spermatogenic cells (**L**). Signal patterns output (**M**) by SPG, pro-Mφ, and SSC cells and signal patterns received (**N**) by them in the iNOA group.

Our previous study conducted a preliminary analysis of scRNA-seq data of testis from an iNOA patient, and the results showed that the imbalance of testicular microenvironment in iNOA patients was closely related to the increase of macrophages and high inflammatory reaction [14], which might be a crucial factor of spermatogenic dysfunction in iNOA patients. This study conducted an in-depth analysis of the scRNA-seq data from iNOA patients and the normal group. The testicular macrophages (CD74+) were extracted for dimensionality reduction clustering and 11 groups of cell types were obtained (Fig. 2A–C). According to the existing reports on Evaluating Inflammatory Score [14, 15], the inflammatory status of each macrophage population was scored (Fig. 2D). Macrophages were further identified and divided into pro-inflammatory macrophages (pro-Mφ) and anti-inflammatory macrophages (anti-Mφ) based on the results of clustering and inflammation score (Fig. 2E). As we suspected, compared with the control group, the number and proportion of pro-Mφ in the testis of iNOA patients was increased significantly, while the proportion of anti-Mφ was decreased (Fig. 2F).

We further analyzed the cellular signal communication between pro-Mφ and spermatogenic cells in the testicular tissue of iNOA patients and the normal groups. In the normal group, except for differentiating spermatogonia (SPG), pro-Mφ performed active intercellular communication with various spermatogenic cells, among which the GRN-SORT1 ligand-receptor had the closest signal communication relationship (Fig. 2G–I). GRN gene mediates inflammatory response by encoding progranulin (PGRN), which performs a significant anti-inflammatory effect [16], thus, this communication relationship suggests that macrophage-mediated immune homeostasis plays an important role in maintaining immune immunity in the normal testicular microenvironment. In iNOA samples, the signal communication between pro-Mφ and differentiating SPG was significantly enhanced, and the most significant changes in the ligand-receptor interaction in signal communication were ANXA1-FPR1 and LGALS9-CD45/CD44, which was related with inflammatory response [17, 18] (Fig. 2J–L).

We analyzed the signals outputted and received by macrophages and SPG. Results showed that in iNOA group, pro-Mφ could communicate with SPG through GALECTIN signals, and communicate with the same kind of macrophages through signals such as CXCL, GALECTIN, COMPLEMENT, ANNEXIN, TNF, and VEGF (Fig. 2M, N). SPG cells could communicate with pro-Mφ through ANNEXIN, VEGF, and CSF signals (Fig. 2M, N). In addition, there was no significant intercellular communication between spermatogonium stem cells (SSC) and pro-Mφ and SPG. These results indicated that macrophages promoted inflammatory reactions through an extensive molecular interaction network in the testicular microenvironment of iNOA patients, which led to the destruction of immune exemption in the testicular microenvironment, thereby affecting the normal spermatogenesis process.

To ascertain whether there was a proinflammatory phenotype of macrophages in the testis of patients with unexplained impaired spermatogenesis, in clinical, seminal samples were obtained from healthy individuals and oligospermia patients for investigation. The clinical examination showed that the sperm antibody expression of these patients was negative (Table S2), the urine test (Table S3) and the blood routine test (Table S4) was normal; however, the sperm counts and motility of oligozoospermia patients was decreased (Table S5).

We isolated sperm from clinical semen and stained for observation. Fluorescent staining results showed that the normal samples (1^#^, 4^#^) exhibited a higher sperm number, and almost no immune cells on the smear, however, in oligospermia samples (47^#^, 29^#^, 9^#^, 19^#^, 10^#^, 26^#^, 52^#^), there was a significant reduction in sperm counts, and an elevated presence of immune cells, including macrophages, granulocytes, and lymphocytes (Supplementary Fig. 2A). The same result was observed in sperm smear by Giemsa staining (Supplementary Fig. 2B). Meanwhile, we determined the inflammatory factors in semen supernatant samples by ELISA. Results showed that the levels of IL-6 and IL-1β in oligospermia samples were elevated compared to those in normal samples (Supplementary Fig. 2C). These results indicated that there was indeed macrophage infiltration in the semen of oligospermia patients, and the increased level of inflammatory factors might be directly related to the occurrence of oligospermia.

### Testis inflammation impairs spermatogenesis

To observe the effects of inflammatory response on spermatogenesis in the testis, we administered LPS to WT mice via intraperitoneal injection, while the control group received PBS injection. LPS can induce systemic inflammatory responses [19], including testicular inflammatory responses. One week later, testis and sperm were collected for detection. The sperm counts and staining results showed that the systemic immune response enhanced by LPS injection resulted in a decrease in mouse sperm counts (Supplementary Fig. 3A, B). Histological examination of testicular tissue revealed mild swelling of the seminiferous tubules in the testis (Supplementary Fig. 3C). The immunofluorescence staining results showed that compared with the control group, systemic intervention with LPS induced the expression of γ-H2AX in spermatogenic cells (Supplementary Fig. 3D), suggesting that the systemic immune inflammatory response induced by LPS led to increased DNA damage in spermatogenic cells. Western blot detection showed that the LPS intervention group had increased expression of pro-Mφ markers CD86 in testicular tissue, and decreased expression of spermatogenic cell markers c-Kit, Plzf (Supplementary Fig. 3E, WB strips in this figure correspond to Supplementary Fig. 3 in WB original data). The qPCR detection showed that the LPS intervention group had increased expression of pro-Mφ markers *IL6* and *IL12b*, decreased expression of anti-Mφ markers *IL10* and *Retnla*, and decreased expression of spermatogenic cell markers *c-Kit* and *Grfα1* (Supplementary Fig. 3F, G).

To verify the effect of pro-Mφ, we extracted peritoneal macrophages from WT mice, and induced macrophage pro-inflammatory polarization with LPS, then injected them into mice testis. After intervention for 3 and 7 days, the testis and sperm were collected for investigation. Multiple immunofluorescences staining showed that the intervention group had increased expression of pro-Mφ markers CD74 and CD86 in testicular tissue (Supplementary Fig. 4A). Pro-Mφ markers *IL6*, *IL12b*, and *NOS2* also increased (Supplementary Fig. 4B). Anti-Mφ markers *Ym1*, *IL10*, and *Retnla* decreased (Supplementary Fig. 4B). Western blot results showed that an increase in pro-Mφ in the testes led to an increase in related markers CD74, CD86 and IFN-γ, while anti-Mφ markers CD163 and CD206 decreased (Supplementary Fig. 4C, D, WB strips in this figure correspond to Supplementary Fig. 4 in WB original data). Fluorescence staining and western blot showed that the increase of pro-inflammatory macrophages caused the increase of γ-H2AX expression in the testis (Supplementary Fig. 4E–H, WB strips in this figure correspond to Supplementary Fig. 4 in WB original data).

Subsequently, the spermatogenic function was detected after injection with pro-Mφ for 3 and 7 days. The results showed that the number of sperm in the pro-Mφ injection group was significantly reduced (Supplementary Fig. 5A, B). Consistently, histological examination showed that the number of SPG and spermatocytes in seminiferous tubules decreased significantly after 7 days of injection (Supplementary Fig. 5C, D). Multiple immunofluorescent staining showed that the expression of SPG and spermatocyte markers Plzf, c-Kit, and scp3 decreased significantly after 7 days of injection (Supplementary Fig. 5E). And the similar results were obtained by qPCR and western blot assay (Supplementary Fig. 5F, G, WB strips in this figure correspond to Supplementary Fig. 5 in WB original data). We also observed that *Grf*α*1* and *Id4* decreased significantly after 7 days of injection (Supplementary Fig. 5F), which indicated that the increase of proinflammatory macrophages in the testis led to spermatogenic dysfunction.

### RNF8 deficiency leads to increased pro-inflammatory polarization of testicular macrophages and affects spermatogenesis

**Fig. 3:**
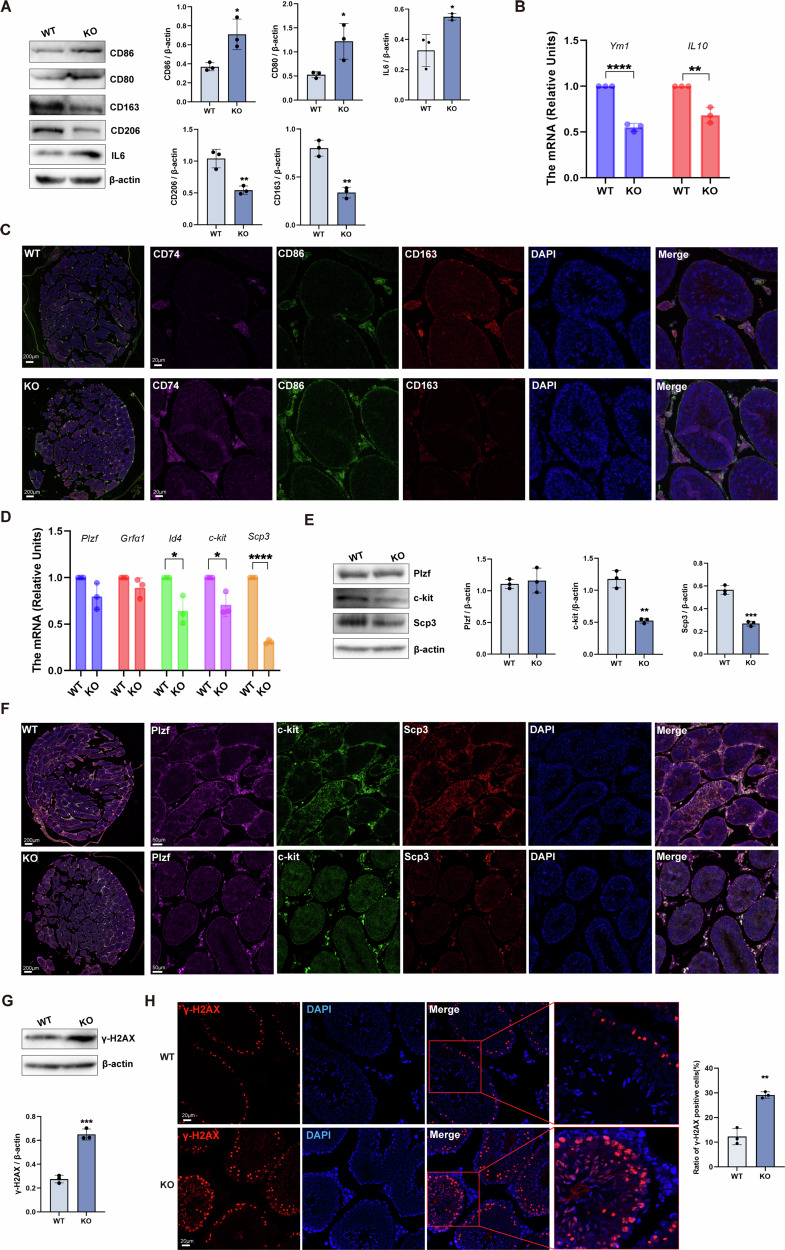
RNF8 deficiency promotes pro-inflammatory polarization of macrophages in the testicular microenvironment and led to spermatogenic dysfunction. **A** The western blot and analysis of pro-Mφ (CD86, CD80, IL6) and anti-Mφ (CD163, CD206) marker protein level in the testis of WT and KO mice. **B** The mRNA of *Ym1* and *IL10* in the testis of WT and KO mice. **C** Multiple immunofluorescences staining of CD74, CD86, and CD163 in the testis of WT and KO groups, scale bar is 200 µm and 20 µm, respectively. **D** Changes of mRNA level of spermatogenic cells in different stages of mice testis in WT and KO groups, including *Plzf*, *Grfα1*, *Id4*, *c-kit*, and *Scp3*. **E** The western blot and analysis of Plzf, c-kit, and Scp3 in WT and KO groups. **F** Multiple immunofluorescences of Plzf, c-kit, and Scp3 in testicles of WT and KO, scale bar is 200 µm and 50 µm, respectively. The western blot (**G**) and immunofluorescence (**H**) were used to detect the expression of γ-H2AX in the testis of WT and KO, scale bar 20 µm. All values were presented as the mean ± SD. Student’s *t*-test; *P < 0.05, **P < 0.01, ***P < 0.001, ****P < 0.0001. WB strips in this figure correspond to Fig. 3 in WB's original data.

Based on the findings of scRNA-seq analysis, we further explored the effect of RNF8 deletion on testicular macrophages. The expression of pro-Mφ markers CD80, CD86, and IL6 in the KO group were increased, while the expression levels of anti-Mφ markers CD163 and CD206 were decreased (Fig. 3A). The qPCR results also performed a decrease in the expression of *Ym1* and *IL10* in the KO group (Fig. 3B). Multiple immunofluorescences staining showed that the expression and distribution of CD74 and CD86 in the testis of KO group was increased, while CD163 expression and distribution was decreased (Fig. 3C). Then the markers of spermatogenic differentiation at different stages were detected. The results showed that the expression and distribution of the markers c-kit (SPG) and Scp3 (spermatocyte) in KO group were decreased (Fig. 3D–F). Meanwhile, the ratio of γ-H2AX-positive cells in the testis of KO mice was increased (Fig. 3G, H), suggesting that the balance of the testicular microenvironment of KO mice was impaired. These results suggested that RNF8 deficiency led to the disorder of the immune microenvironment in the testis, which was characterized by abolished immune privilege and enhanced inflammatory response, which further led to the impairment of spermatogenic function in mice.

To verify that RNF8 deficiency induced increased pro-inflammatory polarization of macrophages, we extracted peritoneal macrophages of RNF8^+/+^ and RNF8^−/−^ mice and injected them into wild-type mice testis, respectively without prior induction by LPS (named WT + WT Mφ group and WT + KO Mφ group) to investigate the effects of RNF8-deficient macrophages on testis. The fluorescent staining, sperm counts, and Giemsa staining showed that compared to the WT + WT Mφ group, the WT + KO Mφ group had more macrophages and lower sperm (Supplementary Fig. 6A–C). The histological examination showed that the number of spermatocytes in WT + KO Mφ group was significantly reduced than that in WT + WT Mφ group (Supplementary Fig. 6D, E). Moreover, the ratio of γ-H2AX positive cells in testis of the WT + KO Mφ group was increased (Supplementary Fig. 6F), suggesting that the damage of spermatogenic cells in this group was more serious.

**Fig. 4:**
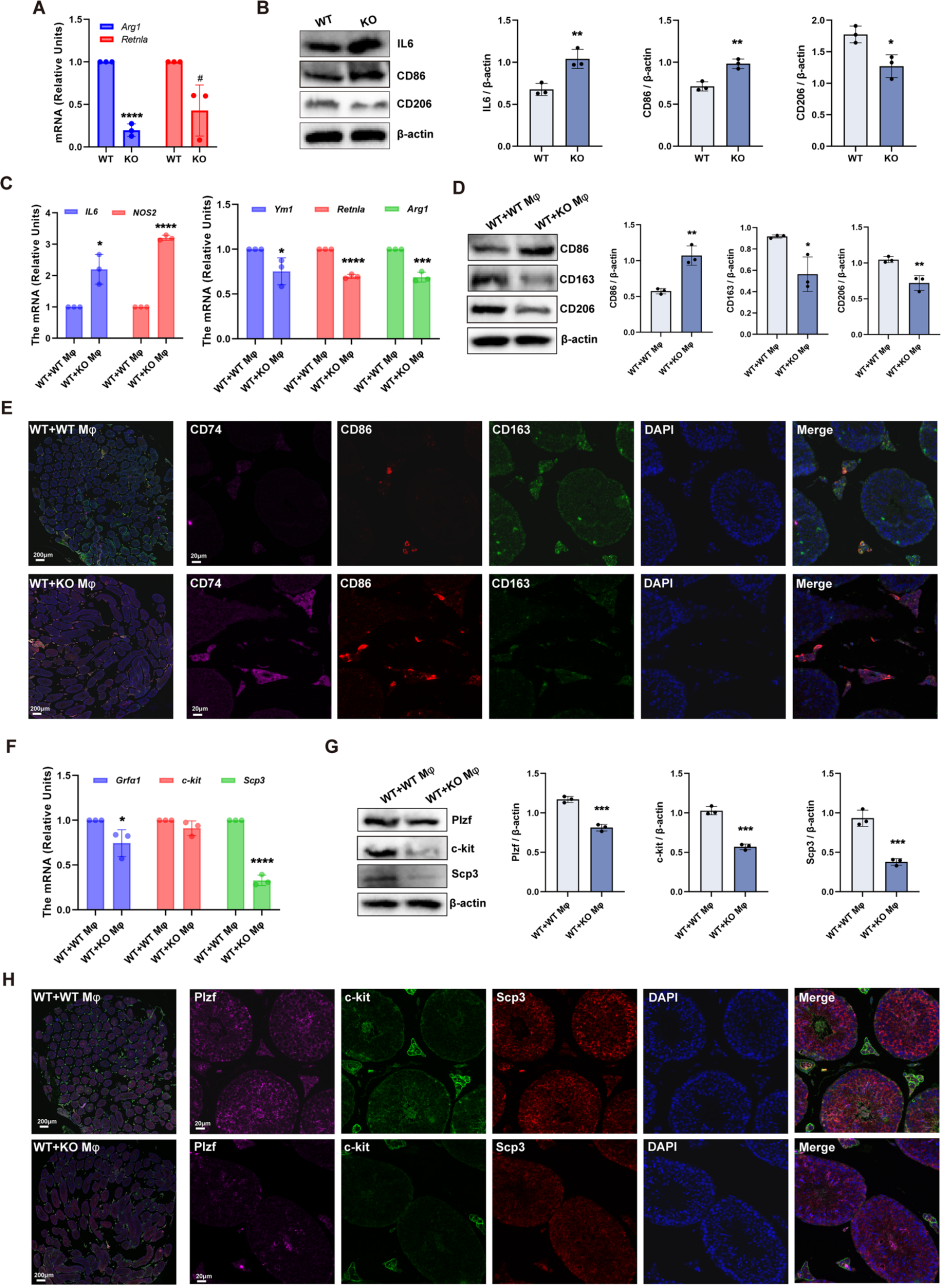
RNF8 deficiency mice peritoneal macrophages hinders the development of spermatogenesis. **A** The mRNA level of anti-Mφ (*Arg1* and *Retnla*) markers in the peritoneal macrophages of WT and KO mice. **B** The protein level of anti-Mφ (CD206) and pro-Mφ (IL6 and CD86) markers in the peritoneal macrophages of WT and KO mice. **C** The mRNA level of *IL6*, *NOS2*, *Ym1*, *Retnla*, and *Arg1* in the testis of WT + WT Mφ group and WT + KO Mφ group. **D** The protein level of CD86, CD163, and CD206 in the testis of WT + WT Mφ group and WT + KO Mφ group. **E** Multiple immunofluorescences staining of CD74, CD86, and CD163 in testicular tissues of the above two groups, scale bar is 200 µm and 20 µm, respectively. **F** The mRNA level spermatogenic cells in different stages (*Grfα1*, *c-kit*, and *Scp3*) of mice testis in the above two groups. **G** The protein level of spermatogenic cells in the testis of the above two groups, including Plzf, c-kit, and Scp3. **H** Multiple immunofluorescences of Plzf, c-kit, and Scp3 in the testis of the above two groups, scale bar is 200 µm and 20 µm, respectively. All values were presented as the mean ± SD. Student’s *t*-test; *P < 0.05, **P < 0.01, ***P < 0.001, ****P < 0.0001. WB strips in this figure correspond to Fig. 4 in WB's original data.

The qPCR and western blot were used to detect the peritoneal macrophages of WT and KO mice. The results showed that the expression of *Arg1*, *Retnla*, and *CD206* decreased in the KO group, while the expression of IL6 and CD86 increased (Fig. 4A, B). Next, we further investigated the immune status and spermatogenic function of the testis. The results of qPCR showed that compared with the WT + WT Mφ group, the expressions of pro-Mφ markers *IL6* and *NOS2* in the testis of the WT + KO Mφ group increased, while the expressions of anti-Mφ markers *Ym1*, *Retnla* and *Arg1* decreased (Fig. 4C). And the results of western blot also confirmed the same conclusion (Fig. 4D). Multiple immunofluorescences staining showed that the expression and distribution of CD74 and CD86 in testis of WT + KO Mφ group was increased, while the CD163 was decreased (Fig. 4E). The qPCR, western blot, and multiple immunofluorescences staining were used to detect the markers related to spermatogenic cell differentiation in each stage. The results showed that *Grfα1* and *Scp3* in the testis of WT + KO Mφ mice decreased (Fig. 4F). Similarly, western blot and multiple immunofluorescences staining indicated that the expression of Plzf, c-kit and Scp3 both decreased in the testis of WT + KO Mφ mice (Fig. 4G, H).

### RNF8 regulates the pro-inflammatory polarization of macrophages through KDM6A-mediated epigenetic modification

**Fig. 5:**
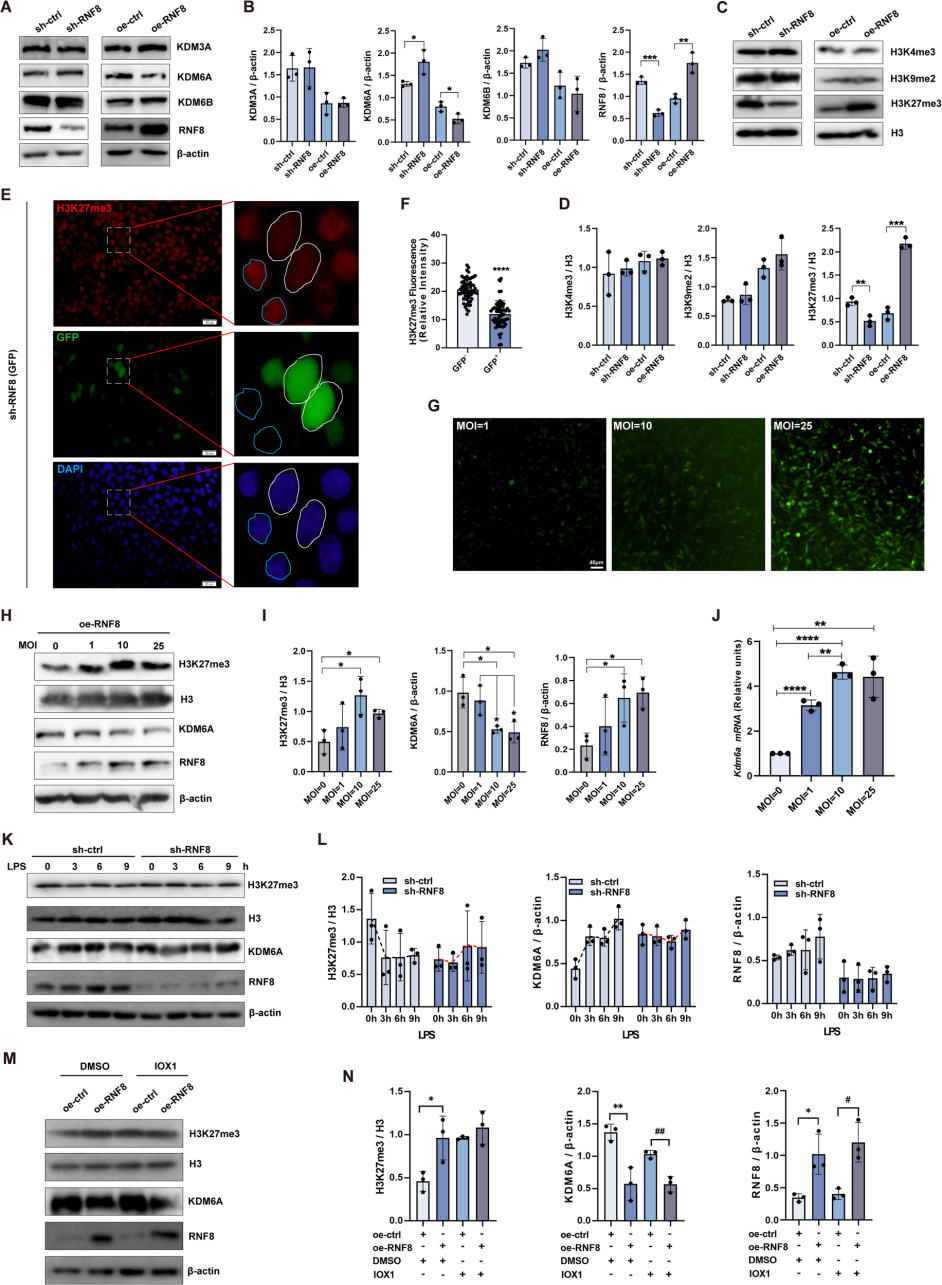
RNF8 regulates the expression of H3K27me3 through KDM6A. **A**–**D** 293T cells knocked down or overexpressed RNF8, and the levels of histone demethylases KDM6A, KDM6B, KDM3A, as well as H3K9me2, K3H4me3, H3K27me3 were detected. **E**, **F** 293T cells knocked down RNF8, and immunofluorescence staining was used to detect the level of H3K27me3 in the successfully knocked down and unsuccessful knocked down cells, *n* = 60. Scale bar 20 µm. Infected cells according to different MOI, and overexpressed RNF8. The transduction efficiency was observed by microscope (**G**), and the change levels of protein (**H**, **I**) of H3K27me3 and KDM6A, and mRNA (**J**) of *Kdm6a* were detected. Scale bar 20 µm. **K**, **L** LPS intervened the sh-ctrl or sh-RNF8 293T cells for 0–9 h, and the protein levels of H3K27me3 and KDM6A were detected by western blot. **M**, **N** After oe-ctrl or oe-RNF8 293T cells were treated with IOX1, the levels of H3K27me3 and KDM6A were detected by western blot. All values were presented as the mean ± SD. Student’s *t*-test; *P < 0.05, **P < 0.01, ***P < 0.001, ****P < 0.0001. WB strips in this figure correspond to Fig. 5 in WB's original data.

Histone methylation plays a key role in maintaining the polarization of macrophages. By dynamically regulating the expression pattern of genes in macrophages, histone methylation can flexibly regulate the polarization of macrophages, which is of great significance for the immune response in the course of autoimmune, psychiatric, and neurodegenerative diseases and cancer [20]. We further examined the effect of RNF8 on histone demethylase. We knocked down or overexpressed RNF8 in 293T cells, and the expression of histone demethylase KDM3A, KDM6A, KDM6B, as well as H3K4me3, H3K9me2, H3K27me3 were detected. The results of western blot showed that RNF8 deficiency led an upregulation of KDM6A; meanwhile, RNF8 overexpression downregulated KDM6A, with no significant alterations trends observed in KDM3A and KDM6B (Fig. 5A, B). For the analysis of H3 methylation, the results indicated a significant decrease in H3K27me3 expression in the RNF8 knockdown group and an increase in the RNF8 overexpressed group (Fig. 5C, D). Immunofluorescence staining revealed that the level of H3K27me3 was decreased when RNF8 was knocked down (Fig. 5E, F).

RNF8 was overexpressed in 293T cells with different MOI (Fig. 5G). The results of western blot showed that the expression of KDM6A was decreased in a concentration-dependent manner concomitant with the elevation of RNF8 expression, and the expression of H3K27me3 was increased (Fig. 5H, I). However, the results of qPCR showed that the mRNA level of *KDM6A* increased with the increase of RNF8 protein expression (Fig. 5J), which was inconsonant with the trend of protein expression in Fig. 5H. And other results showed that the mRNA level of *KDM6A* in the macrophages of the KO group was decreased, but its protein level was increased (Supplementary Fig. 7A–C, WB strips in this figure correspond to Supplementary Fig. 7 in WB original data.). Consistent with this, the H3K27me3 level in the KO group macrophages decreased (Supplementary Fig. 7A–C). These results suggested that the regulation of KDM6A expression might occur at the protein level rather than the transcription level when the expression of RNF8 increased.

We then used LPS to intervene the control and RNF8 knockdown 293T cells, and detected the levels of H3K27me3 and KDM6A at different time points. The results showed that the protein level of H3K27me3 in the control group decreased rapidly and the KDM6A increased from 3 to 9 h after LPS intervention, while the deletion of RNF8 abolished the expression changes of H3K27me3 and KDM6A after LPS intervention (Fig. 5K, L). Then, the histone demethylase inhibitor IOX1 intervention was administered in the case of RNF8 overexpression. Results showed that RNF8 overexpression led to an increase in H3K27me3 expression and a decrease in KDM6A expression, but the IOX1 intervention abolished the expression changes (Fig. 5M, N), indicating that RNF8 regulated the expression of KDM6A protein, thereby affecting the levels of H3K27me3.

Subsequently, we analyzed the expression of KDM6A in the testis and the correlation between KDM6A expression and inflammatory factors. Western blot showed an increase in KDM6A expression in the testis of KO mice (Supplementary Fig. 7D). The immunofluorescence staining performed that high level of KDM6A was mainly distributed in the peripheral layer of seminiferous tubule in KO group (Supplementary Fig. 7E). Researchers analyzed testicular macrophages by spatial transcriptome and single-cell sequencing and proposed a subset named peritubular macrophages. This group of cells is mainly located in the myoid layer adjacent to the SPG cells, highly expresses MHCII molecules, and produces inflammatory mediators to promote the occurrence of inflammatory response [12, 21]. In addition, GTEx database analysis showed that the expression of KDM6A was positively correlated with the expression of pro-inflammatory factors IL6, IL12a, and TNF in the testis and other tissues (Supplementary Fig. 7F), but was negatively correlated with the anti-inflammatory factor IL10 (Supplementary Fig. 7G), indicating that the high level of KDM6A promoted the expression of pro-inflammatory factors. These results indicated that RNF8 affected macrophage pro-inflammatory polarization by regulating the KDM6A-mediated epigenetic modification.

### RNF8 regulates degradation of KDM6A through the autophagy-lysosome pathway

**Fig. 6:**
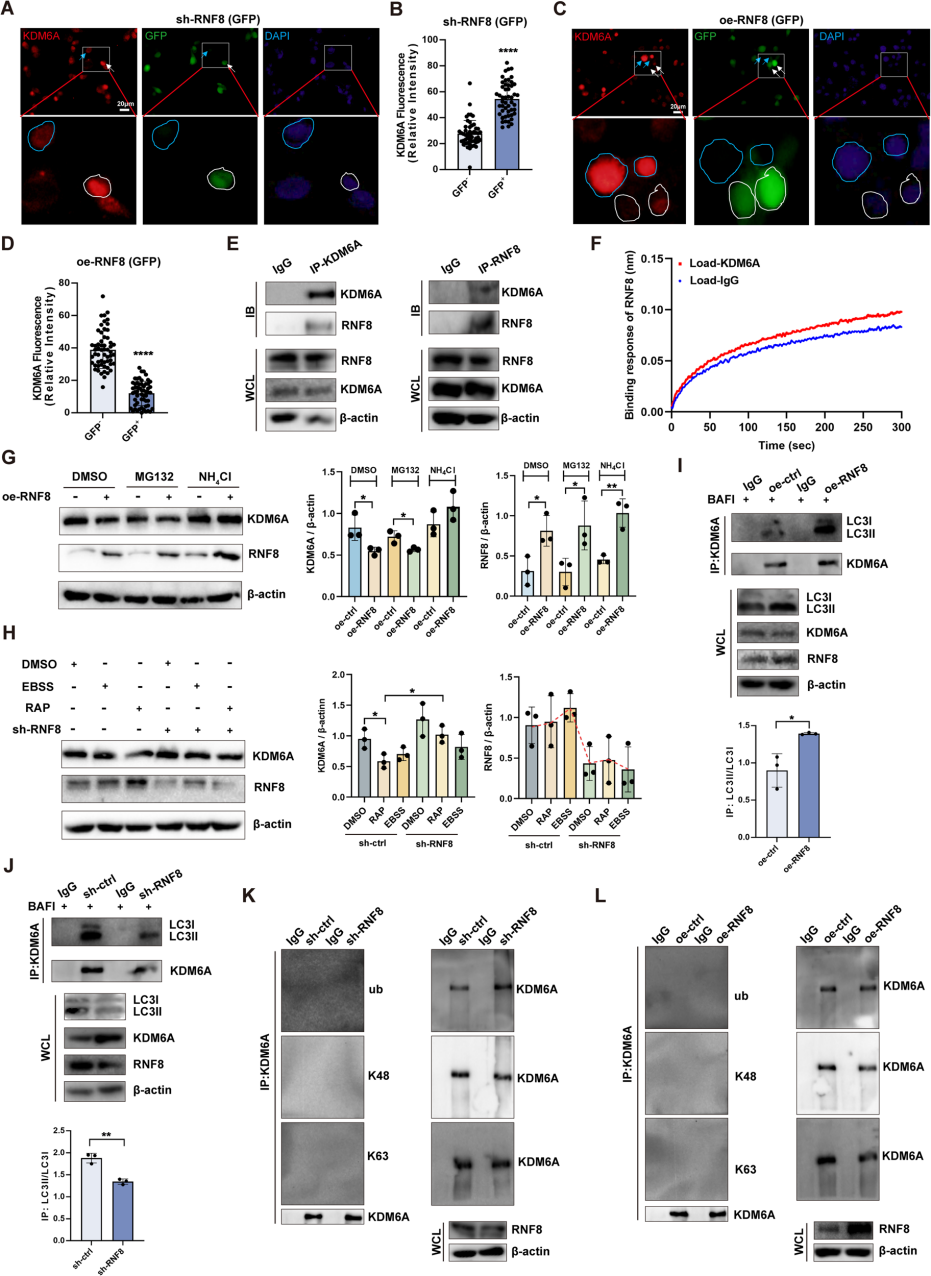
RNF8 regulates the degradation of KDM6A through the autophagy-lysosome pathway. **A**, **B** Detected and statistically analyzed the expression levels of KDM6A in sh-RNF8 293T cells with successful transduction (white arrow) and unsuccessful transduction (blue arrow), *n* = 50. Scale bar 20 µm. **C**, **D** And KDM6A expression levels were detected and statistically analyzed in oe-RNF8 293T cells with successful transduction (white arrow) and unsuccessful transduction (blue arrow), *n* = 57. Scale bar 20 µm. **E** Co-IP detection of KDM6A and RNF8 in 293T cells. **F** Binding response (nm) between KDM6A and RNF8 was measured by Octet R2 system. **G** 10 μM MG132 and 1 mM NH_4_Cl were treated with control 293T cells or oe-RNF8 293T cells for 6 h, respectively, to detect the protein degradation of KDM6A by western blot. **H** EBSS or 100 nM Rapamycin was used to treat 293T cells in the control group or knocked down the RNF8 group for 4 h, respectively, and the expression of KDM6A was detected by western blot. **I**, **J** 293T cells overexpressed or knocked down RNF8, IP-KDM6A, and detected the level changes of LC3I and LC3II enriched by KDM6A. **K**, **L** Knocked down or overexpressed RNF8, IP-KDM6A, and detected the ubiquitin, K48-linked ubiquitination, and K63-linked ubiquitination level enriched by KDM6A. All values were presented as the mean ± SD. Student’s *t*-test; *P < 0.05, **P < 0.01, ****P < 0.0001. WB strips in this figure correspond to Fig. 6 in WB's original data.

Next, we further explored the mechanism of how RNF8 regulated KDM6A. Immunofluorescence staining showed that KDM6A protein expression increased when RNF8 was knocked down in cells (Fig. 6A, B), but decreased when RNF8 was overexpressed (Fig. 6C, D), indicating that KDM6A expression was negatively regulated by RNF8. Co-IP detection showed that RNF8 could bind with KDM6A to form a complex (Fig. 6E). Furthermore, binding between KDM6A and RNF8 was measured in an Octet R2 system, which was based on the biolayer interferometry technology. The result of the real-time analysis showed that there was a specific interaction between KDM6A and RNF8 compared with IgG (Fig. 6F). Protein degradation is mainly through ubiquitination, proteasome, and autophagic lysosome pathways. Under the condition of RNF8 overexpression in cells, the level of KDM6A expression still decreased after intervention with proteasome inhibitor MG132, but remained unchanged after intervention with autophagy inhibitor NH_4_Cl (Fig. 6G). Moreover, we found that RNF8 expression was increased and KDM6A was decreased when the autophagy was induced by Earle’s balanced salt solution (EBSS) or rapamycin (RAP), but RNF8 knockdown reversed the decrease in KDM6A (Fig. 6H).

Subsequently, we overexpressed or knocked down RNF8 in the cells, and co-IP results showed that KDM6A could bind with LC3, and the binding capacity increased when RNF8 was overexpressed (Fig. 6I) and decreased when RNF8 was knocked down (Fig. 6J), suggesting that RNF8 might promote the degradation of KDM6A through autophagy. We examined whether RNF8 performed a ubiquitination effect on KDM6A. The results showed that the ubiquitination, K48-linked ubiquitination, and K63-linked ubiquitination level of KDM6A did not change when RNF8 was overexpressed or knocked down (Fig. 6K, L). These results illustrated that KDM6A was degraded through the autophagy-lysosome pathway rather than ubiquitin-proteasome pathway mediated by RNF8, although RNF8 bond to KDM6A.

Further, we verified the relationship between RNF8 and autophagy-related proteins. Autophagy was induced by EBSS, and the expression and co-location distribution of RNF8, OPTN, p62, and LC3II during autophagy were detected. Immunofluorescence staining showed that RNF8 could be translocated from the nucleus to the cytoplasm during autophagy, and co-localized with OPTN, p62, and LC3II (Supplementary Fig. 8).

We also investigated the expression changes of RNF8, OPTN, p62, and LC3II in the cytoplasm and nucleus of 293T cells during autophagy. After EBSS intervention, the expression of p62 and OPTN increased within 6 h and then decreased at 12 and 24 h (Supplementary Fig. 9A, B, WB strips in this figure correspond to Supplementary Fig. 9 in WB original data). We then examined the protein changes within 6 h of the EBSS intervention, and a large number of RNF8 in the nucleus was translocated to the cytoplasm. The expression of OPTN, p62, and LC3II increased significantly (Supplementary Fig. 9C, D, WB strips in this figure correspond to Supplementary Fig. 9 in WB original data). Similarly, when using RAP intervention to induce autophagy for 6 h, the expression changes of the proteins were consistent with the trend of EBSS experimental results (Supplementary Fig. 9E, F, WB strips in this figure correspond to Supplementary Fig. 9 in WB original data). Then, 293T cells were transfected with HA-OPTN, and after 4 h of intervention with RAP, the levels of ub and K63 of OPTN increased, while there was no significant change in K48 levels (Supplementary Fig. 9G, WB strips in this figure correspond to Supplementary Fig. 9 in WB original data), indicating that OPTN could be ubiquitinated during autophagy, with K63-linked ubiquitination being the main type of modification.

### RNF8 decreases KDM6A by regulating ubiquitination of OPTN

**Fig. 7:**
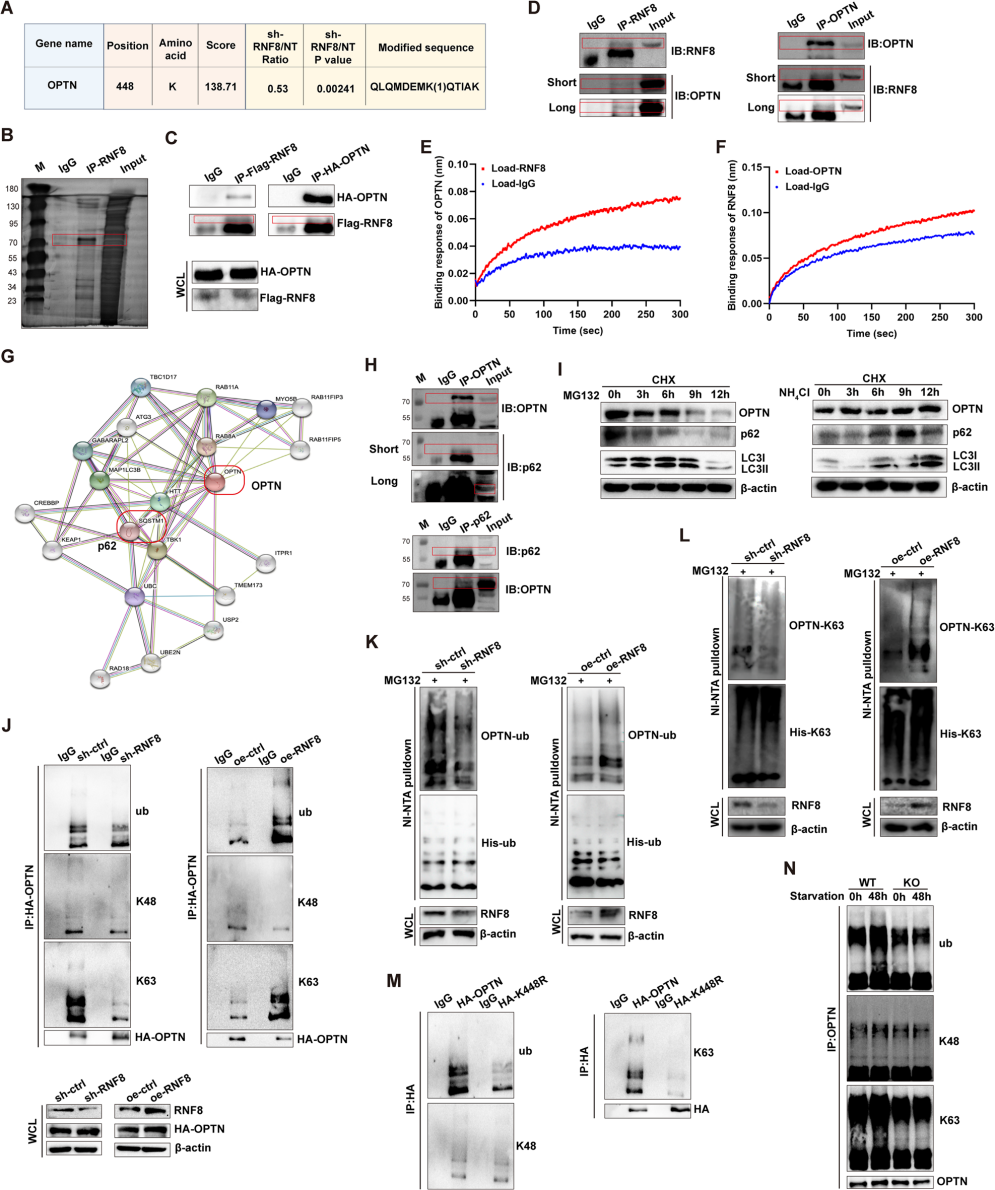
RNF8 regulates the activity of OPTN by mediating K63 ubiquitination modification. **A** RNF8 ubiquitination modification omics data. **B** IP-RNF8 in 293T cells, silver stained. Exogenous (**C**) and endogenous (**D**) co-IP detection of RNF8 and OPTN. **E**, **F** Binding response (nm) between RNF8 and OPTN was measured by Octet R2 system. **G** The interaction between OPTN and SQSTM1 (p62) in the STRING database. **H** Endogenous co-IP results of OPTN and p62. **I** After treatment with MG132 or NH_4_Cl, the expression changes of OPTN, p62, and LC3 were detected. **J** HA-OPTN was transfected into 293T cells with knockdown or overexpression of RNF8, and its ubiquitin level was detected by IP-HA. **K** Transfected His-ub in 293T cells with knockdown or overexpression of RNF8, and detected the change of ubiquitin level of OPTN. **L** Transfected His-K63 in 293T cells with knockdown or overexpression of RNF8, and detected the change of K63 ubiquitin level of OPTN. **M** Transfected wild-type OPTN or K448R into 293T cells and detected the change of ubiquitin levels of OPTN. **N** After 48 h of starvation treatment in WT or KO mice, the changes in ubiquitin levels of OPTN in testicular tissue were detected. WB strips in this figure correspond to Fig. 7 in WB's original data.

The ubiquitination modification omics showed that OPTN could be ubiquitinated on K448 in 293T cells, where the level of OPTN ubiquitination was significantly decreased in KO group than in control group (Fig. 7A). After immunoprecipitation of RNF8, gel electrophoresis and silver staining were performed, and an obvious protein band was observed near 70 KD (Fig. 7B). Next, we validated the interaction between RNF8 and OPTN, as well as the impact of RNF8 on autophagy progression. The endogenous and exogenous co-IP assay showed that RNF8 could interact with OPTN (Fig. 7C, D). And the binding between RNF8 and OPTN was interacted in an Octet R2 system (Fig. 7E, F). It has been reported that the ubiquitination of OPTN promotes its interaction with p62/SQSTM1 to form autophagy receptor complex, thus accelerating autophagy flux [22]. Through the validation of the STRING database and co-IP, the results showed that OPTN and p62 can form a complex (Fig. 7G, H). Using MG132 and cycloheximide (CHX) intervention, the degradation of OPTN and related autophagic proteins at different time points was detected. The results showed that the expression of OPTN, p62, and LC3 decreased with time. With the intervention of autophagy inhibitors NH_4_Cl and CHX, it was shown that under the condition of autophagy inhibition, the expression of OPTN, p62, and LC3 increased with time (Fig. 7I), indicating that the degradation of OPTN and its related autophagic proteins is not affected by changes in the proteasome pathway, but is degraded through the autophagy-lysosome pathway.

After knocking down RNF8 in 293T cells, the ub and K63 levels of OPTN decreased, but the K48 level did not change significantly. After overexpression of RNF8, ub and K63 ubiquitin levels of OPTN increased, while K48 did not change significantly (Fig. 7J). Ni^2+^ pull-down assay was used to detect the ubiquitin level of OPTN when RNF8 was knocked down or overexpressed. The results showed that His-ub (Fig. 7K) and His-K63 (Fig. 7L) of OPTN decreased when RNF8 was knocked down. His-ub (Fig. 7K) and His-K63 (Fig. 7L) of OPTN increased when RNF8 was overexpressed. Subsequently, we mutated the K448 site of OPTN to R, and exogenous transfected HA-OPTN or HA-OPTN-K448R. The results showed that ub and K63 levels of OPTN in the K448R group decreased (Fig. 7M), indicating that RNF8 modified OPTN with K63-linked ubiquitination to regulate its activity, and the modification site was K448. Meanwhile, in KO mice induced autophagy by starvation treatment, the levels of ub and K63 ubiquitin in the testicular tissue of OPTN did not increase, while the levels of ub and K63 in the testicular tissue of WT mice increased after starvation treatment (Fig. 7N), indicating that RNF8 deficiency affected the K63-ub level of OPTN and thus affects autophagy function.

**Fig. 8:**
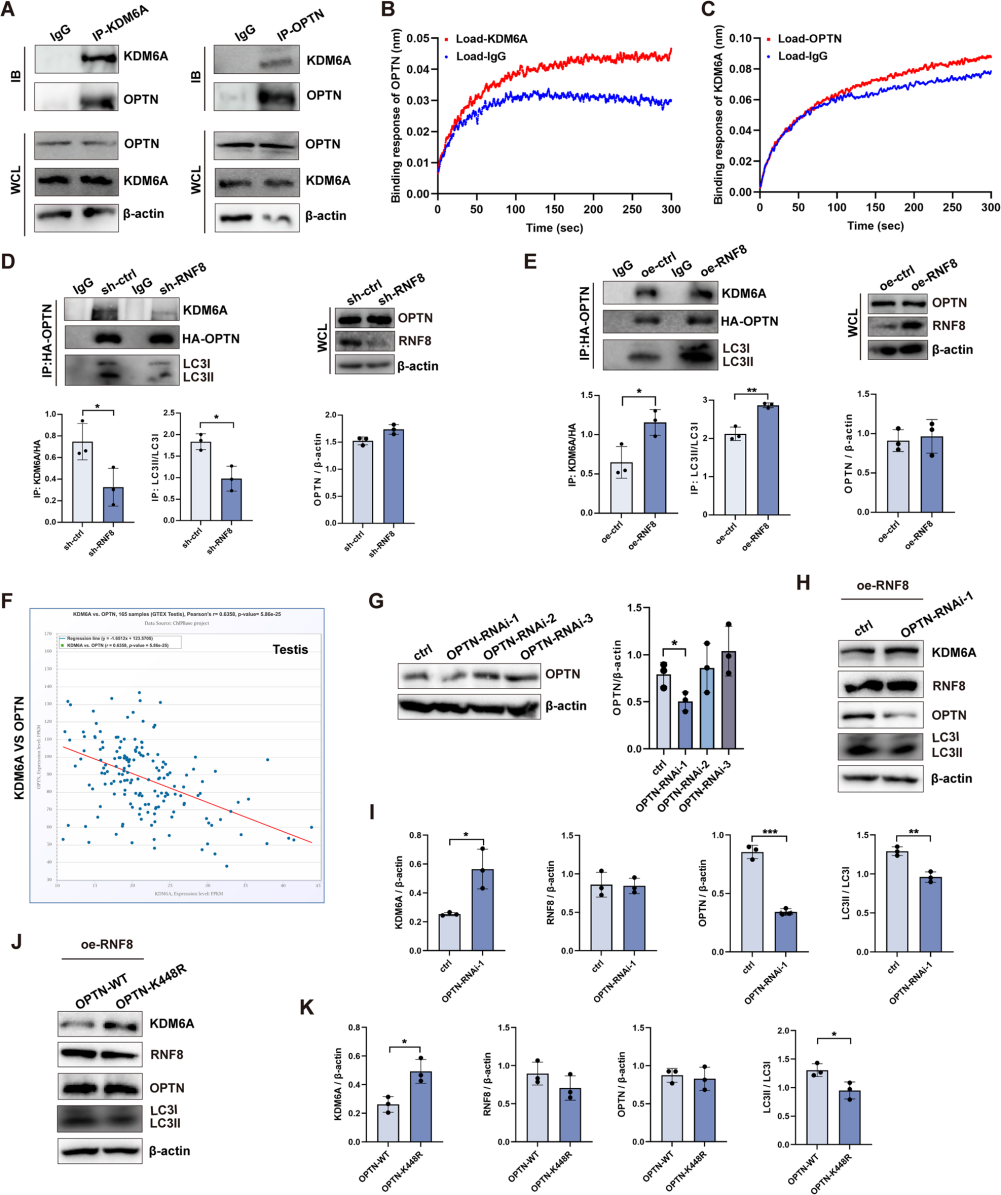
RNF8 regulates the degradation of KDM6A through ubiquitination OPTN. **A** Co-IP detection of the interaction between KDM6A and OPTN in cells. **B**, **C** Binding response (nm) between KDM6A and OPTN was measured by Octet R2 system. **D**, **E** 293T knocked down or overexpressed RNF8, IP-HA-OPTN, and detected the expression changes of KDM6A and LC3II. **F** GTEX database was used to analyze the correlation between the expression of OPTN and KDM6A in the testis. **G** Verification of OPTN knockout plasmid. **H**, **I** knocked down OPTN in 293T cells overexpressing RNF8 and detected the changes of KDM6A and LC3. **J**, **K** K448R was transfected into 293T cells overexpressing RNF8, and the changes of KDM6A and LC3 were detected. All values were presented as the mean ± SD. Student’s *t*-test; *P < 0.05, **P < 0.01, ***P < 0.001.WB strips in this figure correspond to Fig. 8 in WB's original data.

Co-IP and binding determination results showed that OPTN and KDM6A could interact to form a complex (Fig. 8A–C). What’s more, knocked down RNF8 led to reduced binding of OPTN to KDM6A and OPTN to LC3II (Fig. 8D). On the contrary, overexpression of RNF8 resulted in increased binding of OPTN to KDM6A and OPTN to LC3II (Fig. 8E), indicating that RNF8 expression was crucial in OPTN-mediated degradation of KDM6A through the autophagy pathway. GTEx database analysis showed that OPTN was negatively correlated with KDM6A expression in testicular tissue (Fig. 8F). Knocked down OPTN in 293T cells and screened the plasmid with high efficiency (Fig. 8G). Knocking down OPTN in 293T cells stably overexpressing RNF8 showed that the expression of KDM6A increased and LC3II decreased (Fig. 8H, I). Subsequently, OPTN-K448R was transfected into 293T cells overexpressing RNF8, and similar results were obtained (Fig. 8J, K). These results indicate that when RNF8 is normally expressed, inhibition of OPTN or mutation of the K448 site of OPTN will affect the degradation of KDM6A. Activation of the K448 site of OPTN is an important prerequisite for the degradation of KDM6A by lysosomal pathway.

## Discussion

During the differentiation and development of SPG into sperm, histones are gradually replaced by transition proteins and fish sperm proteins, forming a dense nuclear structure that achieves high chromatin compression to maintain effective transmission of genetic information from sperm to offspring during fertilization [23]. MIWI is highly expressed in the testes and is an essential gene for male fertility [24]. It has been suggested that MIWI is separated from RNF8, a key E3 ligase for histone ubiquitination in the early sperm cytoplasm and that APC/C degradation of MIWI in later sperm cells releases RNF8 into the nucleus to mediate histone ubiquitination and subsequent histone/protamine exchange [25]. RNF8 is an E3 ubiquitin ligase that participates in the regulation of biological processes such as protein ubiquitination modification, DNA damage repair, and genome stability in cells [25, 26]. Our previous studies have shown that RNF8 is critical in tumor progression, treatment, and neurodegeneration [3, 27–29]. Our study with Lu et al. found that RNF8 gene knockout mice exhibited male infertility phenotype [3, 4]. We speculated that RNF8 deficiency led to defect in histone ubiquitination modification, which may hinder the replacement process of histone into transition proteins and protamine, resulting in an increase in abnormal sperm. But soon Hironori Abe et al. proposed that RNF8 was not necessary for histone replacement as a transition protein and protamine, and provided experimental evidence for the expression of transition proteins and protamine in RNF8−/− mouse spermatogenic cells [5]. These results brought controversy and challenges to explore the mechanism of male infertility phenotype in RNF8 gene-deficient mice. Through scRNA-seq analysis of RNF8^+/+^ and RNF8^−/−^ mouse testes, we found that RNF8 deficiency leads to delayed spermatogenesis, impaired differentiation of spermatocytes and spermatocytes, and increased macrophages and T cells (Fig. 1C). The analysis showed that the sperm differentiation process of KO mice was mainly blocked at the spermatocyte stage (Fig. 1F) and KO mice showed reduced expression of germ cell markers (Supplementary Fig. 1A, B).

Systemic tolerance and local immune privilege are prerequisites for testicular immune protection. Antigen-specific regulatory T cells continuously export germ cell antigens through the endocytosis of Sertoli cells, maintaining systemic tolerance in peripheral lymphatic organs. Leydig cells, macrophages, Sertoli cells, peritubular cells, and the blood-testis barrier in the testes play an important role in maintaining the homeostasis of the testicular microenvironment. Under physiological conditions, low levels of pro-inflammatory factors in the microenvironment are crucial for maintaining normal spermatogenesis. However, enhanced immune responses can lead to spermatogenic dysfunction and germ cell apoptosis [6, 7]. In the response of immune cells to autoantigens, the activation of lymphomonocytes may trigger the release of chemokines, cytokines, and reactive oxygen species (ROS), leading to obstruction of spermatogenesis [30].

The levels and activation of macrophages and dendritic cells in patients with chronic genital tract inflammation are closely related to oligospermia and asthenospermia [31]. Abnormal spermatogenesis in patients with azoospermia is associated with increased numbers of macrophages, T cells, B cells, and mast cells, and the inflammatory reaction in the testis is enlarged [32]. The reduction of T cells in the testis does not affect testicular development and spermatogenesis, and normal differentiated germ cell populations can still be developed, while the increase of antigen-presenting cells and their mediated T cell accumulation are directly related to the occurrence of chronic autoimmune inflammation of the testis. The increase of IL6, IL1, and other cytokines has also been confirmed to have a clear damage effect on the structure and function of testis tissue [33, 34]. Anti-inflammatory therapy has been demonstrated to contribute to sperm production and fertility recovery [35]. These results suggest that immune cell-mediated hyperinflammation leads to the disruption of immune homeostasis in the testicular microenvironment, which ultimately leads to reduced fertility. We collected clinical samples and found that the expression of IL-6 and IL-1β was increased in the semen supernatant of oligospermia patients (SupSupplementary Fig. 5). The scRNA-seq analysis of clinical samples from iNOA patients revealed a significant increase in the proportion of pro-inflammatory macrophages and signal communication with spermatogenic cells in the testis compared with the control group (Fig. 2). Our study showed an increase in T cells and macrophages (Fig. 1C) and an enhanced immune inflammatory response (Fig. 1E) in the testes of KO mice compared to the control group.

At present, in a few studies, testicular macrophages are classified into interstitial macrophages and peritubular macrophages through scRNA-seq, spatial transcriptomics, and flow cytometry analysis. It is considered that they have different surface marker expressions, spatial distributions, cell morphology, and function [21, 36]. These views suggest that interstitial macrophages are mainly distributed in the interstitial space, highly expressing immunosuppression-related genes, while peritubular macrophages are located in the myoid layer adjacent to SPG, highly expressing antigen presentation-related genes such as MHCII, and can produce inflammatory mediators such as IL1 and CXCR4. The high expression of MHCII can activate regulatory T cells and regulate the continuous occurrence of the immune response through antigen presentation [37, 38]. These two cell populations play distinct but complementary roles in maintaining immune homeostasis within the testicular microenvironment, creating a favorable environment for spermatogenesis by maintaining a balance between pro-inflammatory and anti-inflammatory mediators [39]. In our study, we did not clearly analyze such macrophage subpopulations from single-cell samples, which may be related to differences in species and the number of testicular cells. However, our data classified testicular macrophages into pro-inflammatory and anti-inflammatory types based on their phenotypic differences. At the same time, we found that the testicular tissue of KO mice showed an increase in pro-inflammatory macrophages and a decrease in anti-inflammatory macrophages (Fig. 3A–C), accompanied by impaired spermatogenesis (Fig. 3D–F). In order to clarify the effect of testicular immune microenvironment remodeling on spermatogenic function, we induced systemic immune response and injected pro-inflammatory macrophages into the testes of mice, confirming that the pro-inflammatory microenvironment in the testes indeed leads to damage to spermatogenic cells and impaired spermatogenic function (Supplementary Figs. 2–4). Furthermore, to demonstrate that the increase of pro-inflammatory macrophages in RNF8 deficiency mice leads to impaired spermatogenic function, we injected peritoneal macrophages from both WT and KO mice into the testes of WT mice. As expected, injection of RNF8-deficient macrophages resulted in significant impairment of spermatogenic function in WT mice (Supplementary Fig. 6 and Fig. 4). These results indicated that the increase in pro-inflammatory macrophage levels was closely related to spermatogenic dysfunction, and RNF8 may play a crucial role in maintaining macrophage polarization and immune stability in the testicular microenvironment.

Autophagy plays an important role in the polarization of macrophages [40]. Inhibition of autophagy induces polarization of macrophages towards a pro-inflammatory phenotype as M1 macrophages, promotes immune response, and causes inflammation in tissues [41]. Conversely, increasing autophagy flux promotes the polarization of macrophages toward an anti-inflammatory phenotype, commonly known as M2 macrophage [42, 43]. OPTN (optineurin) plays roles in protein trafficking, autophagy, and NFκB signaling activation. Evidently, OPTN has been identified as an autophagy receptor connecting the autophagy substrates with LC3 [44], and also an autophagy inducer that initiates the autophagic process [45]. Moreover, studies have suggested that OPTN plays a variety of functions during autophagy, including initiation and biogenesis of phagophore, interaction with LC3, and maturation of autophagosome [46–49].

OPTN can be regulated through both the autophagy lysosomal pathway and the ubiquitin-proteasome system [22, 50], in which Hrd1 acts as an E3 ubiquitin ligase responsible for the ubiquitylation and degradation of OPTN [50]. Our study indicated that RNF8 assembled the K63-linked ub chain on OPTN, and affected the localization of OPTN, p62, and LC3II on autophagosome, thus mediating autophagy flux (Fig. 7 and Supplementary Figs. 8 and 9). Moreover, mass spectrometry detection revealed that the ubiquitination modification site of OPTN was K448, and the mutation of K448 eliminated the ubiquitination modification of RNF8 on OPTN (Fig. 7). What’s more, our study was also the first to elucidate the essential role of RNF8 in regulating autophagy.

The histone methylation plays an important role in the polarization switching of macrophages by activating or inhibiting the transcriptional expression of downstream factors. KDM6A is responsible for removing dimethyl and trimethyl from histone 3 lysine 27 (H3k27) and activating the transcription of the target gene [51]. Due to H3K27me3 being one of the key histone modifications involved in transcriptional inhibition [52], KDM6A plays a crucial role in inflammatory response [53] and cell differentiation [54]. After LPS stimulation of macrophages, KDM6A promotes the expression of IL6 by demethylating H3K27me3 at the IL6 promoter, and facilitates IFNβ expression in LPS-induced macrophages through interaction with MLL4, promoting polarization towards M1-type macrophages [55]. GSK-J4, an inhibitor of KDM6A, can increase the expression of H3K27me3 in macrophages, leading to the down-regulation of CCL8, CXCL9, CXCL10, and CXCL11, which affects the pro-inflammatory polarization of macrophages [56]. KDM6A ablation activated cytokine and chemokine pathways, promoted M2 macrophage polarization [53], and alleviated the retinal inflammation in diabetic mice [57]. We found that KDM6A was highly expressed in peritoneal macrophages of KO mice (Supplementary Fig. 7B, C), and we speculated that the pro-inflammatory polarization of macrophages caused by RNF8 deficiency may be related to the upregulation of KDM6A expression. Given the reported relationship between KDM6A and macrophages [55, 58], our analysis found that KDM6A was positively correlated with pro-inflammatory factors IL6 and IL12a, and negatively correlated with anti-inflammatory factor IL10 (Supplementary Fig. 7F, G), indicating that high expression of KDM6A mediated the polarization of macrophages towards a pro-inflammatory phenotype.

In conclusion, we revealed a specific molecular mechanism involved in how RNF8 deficiency contributed to spermatogenic dysfunction that different from current studies for the first time. RNF8 affected spermatogenic cells by regulating immune responses, rather than by impacting protamine assembly directly. We proposed that the destruction of immune privilege in testicular microenvironment played a crucial role in spermatogenic function impairment. What’s more, our findings provided novel insights into male sterility and may present novel clinical therapeutic strategies for spermatogenic dysfunction caused by immune disorders.

## Methods

### Animals

The RNF8-deficient mice were provided by Xiaochun Yu and have been previously described [4]. This study used 2-month-old RNF8^−/−^ and RNF8^+/+^ male 129S mice constructed earlier by the team. All animals were maintained in the SPF-level laboratory of Lanzhou University Animal Center and were fed under standard conditions with adequate access to food and water. The experiment plan was approved by the animal ethics committee of Lanzhou University. The ethical approval number is jcyxy20220207.

### Cells lines and cell culture

293T cells (CL-0005, Pricella) was obtained directly from Procell, Wuhan, China. 293T cells were cultured at 37 °C with 5% CO_2_ in complete DMEM medium (HyClone, SH30022.01) supplemented with 10% FBS (Gibco, 10100147 C) and 1% penicillin/streptomycin (Solarbio, P1400). Primary peritoneal macrophages were cultured at 37 °C with 5% CO_2_ in complete 1640 medium (Cytiva, SH30809.01) supplemented with 10% FBS and 1% penicillin/streptomycin.

### Samples of oligozoospermia patients

Semen samples were taken from the normal population and oligozoospermia patients (6 normal individuals and 10 oligozoospermia patients) at the First Affiliated Hospital of Lanzhou University, and all patients signed informed consent forms. The study was approved by the Ethics Committee of Lanzhou University (Lzujcyxy20240327).

### The scRNA-seq by 10×Genomics

The sequencing was provided by OE Biotech Co., Ltd (Shanghai, China). The scRNA-seq libraries were prepared using 10×Genomics Chromium Single Cell 3′ Reagent Kits. Briefly, FACS(Fluorescence-activated Cell Sorting)-sorted cells were resuspended to a final cell concentration of 700–1200 cells µL^−1^ with more than 85% viability as determined by Countess II (Thermo Fisher Scientific). 8000–12,000 cells were captured in droplets. After the reverse transcription step, emulsions were broken and barcoded cDNA was purified with Dynabeads, followed by PCR amplification. Amplified cDNA was then used for 3′ gene expression library construction. Fifty nanograms of amplified cDNA were fragmented and end-repaired. DNA fragmentation was analyzed by Fragment Analyzer 5300 (Aglient), double-size selected with SPRI select beads (avg. size 450 bp), and sequenced on an Illumina platform using 150 paired-end reads at a coverage of 40,000 mean reads per cell.

### The scRNA-seq data processing

The scRNA-seq data analysis was conducted using the Seurat (v4.3.0) in the R environment (v.4.0.2). For quality control, cells were filtered by gene numbers (<200), UMI (Unique Molecular Identifier) (<1000), log10GenesPerUMI (<0.7), percentage of mitochondrial RNA UMIs (>10%) and percentage of hemoglobin RNA UMIs (>5%). The remaining cells underwent downstream analysis, which included normalization, selection of highly variable genes, scaling, and principal component analysis (PCA). To eliminate the batch effect of our combined scRNA-seq data, we utilized the harmony package in accordance with its official guidelines. Following the harmony integration process, we performed uniform manifold approximation and projection (UMAP) via the RunUMAP function. Cell clusters were identified using the FindNeighbors and FindClusters (resolution = 0.4) functions. All standard analysis procedures were conducted using default parameters.

### GSEA and trajectory analysis

To identify any differences between the RNF8^+/+^ (WT) and RNF8^−/−^ (KO) groups, we performed a gene expression analysis. The criteria for determining differential gene expression were based on a threshold of absolute log2 fold change (FC) > 1 and adjusted *p* value < 0.01. We sorted the average log2FC for gene set enrichment analysis (GSEA) using the clusterProfiler package. The results were visualized using the dotplotGsea function.

To investigate the disparities in the evolution of SPG between the RNF8^+/+^ (WT) and RNF8^−/−^ (KO) groups, we conducted a trajectory analysis utilizing the Monocle2 package and deducing the trajectory of targeted cells by implementing a dimensionality reduction technique to arrange the cells in pseudotime. The plot_cell_trajectory function was employed to visualize the results.

### Acquisition of iNOA (idiopathic non-obstructive azoospermia) patients' data

The scRNA-seq data for this study was obtained from the Gene Expression Omnibus database [59, 60], which includes five normal samples and three iNOA samples in GSE149512, as well as three iNOA samples in GSE154535, totaling 11 [14].

### Cell–cell interaction/communication analysis

To gain a comprehensive understanding of the cell–cell interactions, we utilized the CellChat package to infer and analyze the communication between cells [61, 62]. By leveraging the ligand–receptor pairs in CellChatDB (http://tcm.zju.edu.cn/celltalkdb), we performed separate analyses of the cell–cell interactions in both the iNOA and the normal groups.

### Evaluating inflammatory score

Using the AddModuleScore function of Seurat software [15], inflammation status scores were performed on various macrophage populations based on the “Hallmark inflammation response” gene set defined in MsigDB (Molecular Signature Database). To assess the gene-set enrichment score for each cell, we utilized three algorithms: AddModuleScore from the Seurat package, single-sample gene set enrichment analysis (ssGSEA) from the GSVA package, and score single cells with gene regulatory networks (AUCell) from the AUCell package.

### Extraction of primary mice peritoneal macrophages

Three days before the start of the experiment, mice were intraperitoneally injected with 1 ml of 3% thioacetate broth once a day. After the mice were euthanized by cervical dislocation, 5 ml of pre-cooled PBS was injected into the lower right abdomen. The abdomen of the mice was gently rubbed to allow the fluid to flow fully in the abdominal cavity. After recovery, it was centrifuged at 1000 r/min for 5 min, and the supernatant was discarded to obtain peritoneal macrophages.

### Extraction of mice sperm

After the mice were euthanized by cervical dislocation, their epididymis was stripped and transferred to PBS. Then, the epididymal tail was stripped and cut into small pieces to release sperm, which were placed in sperm preservation solution.

### Mice testicular hyperinflammation model

According to the injection method in the testicles by Ani Chi et al. [63], we injected macrophages into the testicles to establish a high inflammation model of the testicles. The peritoneal macrophages of wild-type mice were collected and induced to pro-inflammatory macrophages by 80 μg/ml LPS (MCE, HY-D1056) for 4 h, and injected into the testis of wild-type mice (1 × 10^3^ macrophages/testis). The “sham surgery” control mice used the same surgical method, injecting 50 µl of sterile physiological saline into the testes, and bonding the wound with animal tissue glue (3 M Vetbond, 1469SB). The testes were taken on the 3rd and 7th day after surgery for subsequent experiments.

The peritoneal macrophages of WT and KO mice were collected and injected directly into the testis of wild-type mice (1 × 10^3^ macrophages/testis), and the wound was bonded with tissue glue. On the seventh day, the testis was taken for follow-up experiments.

### AKOYA opal 4-color manual IHC assay

After antigen repair, the paraffin sections of mice testicular tissue were blocked at room temperature for 10 min, and the primary antibody was incubated overnight at 4 °C. PBST was washed three times, and the secondary antibody was incubated at room temperature for 10 min. Opal dye (Marlborough, 211229014) was incubated in the dark for 10 min. The second and third rounds of staining were repeated according to the above steps. Finally, an appropriate amount of DAPI staining was added for 5 min. After sealing, the slides were scanned and photographed using a spatial phenotype analyzer.

### Immunofluorescence

After dewaxing and rehydrating the paraffin sections of mice testis and repairing the tissue antigen, they were blocked with 10% goat serum (Solarbio, S9070) at room temperature for 30 min, washed with TBST three times, incubated with primary antibody overnight at 4 °C, washed with TBST, incubated with secondary antibody Cy3-anti-rabbit (Invitrogen, A10520) at room temperature for 1 h. DAPI (Solarbio, 28718-90-3) was added dropwise for sealing.

After the cell slides were fixed with 4%PA, the membrane was broken with 1% TritonX-100 for 10 min, and the steps after TBST washing were the same as above. The fluorescent secondary antibody used in this part was: FITC-anti-rabbit (Invitrogen, 65-6111), Cy3-anti-rabbit (Abcam, ab6939), Cy3-anti-mice (Invitrogen, A10521).

### Co-IP

The 293T cells were lysed by RIPA, and the supernatant was collected after centrifugation. Protein A/G magnetic beads (BeaverBeads protein A Immunoprecipitation Kit, 22202-20) and 5 mg/ml antibody were added and incubated at 4 °C for 4 h. The cells were washed three times with IP buffer. After denaturation and elution, the precipitate components were analyzed using SDS-PAGE and transferred to the PVDF membrane for analysis. In the IP ubiquitin experiment, additional proteasome inhibitors (MG132, HY-13259, MCE; Epoxomicin, HY-13821, MCE) and deubiquitinase inhibitors (PR-619, HY-13814, MCE) need to be added.

### NI^2+^ pull-down experiment

Transfect His ubi or His ubi-K63 plasmids in 293T cells with stable knockdown or overexpression of RNF8. Collect cells and add Ni^2+^ pull down lysis solution until all precipitates disappear. Add nickel column material (QIAGEN, 172034196) and incubate at room temperature for 4 h. Wash 4 times with Ni^2+^ pull down rinse solution, add Ni^2+^ pull down eluent, mix well, and let it stand at room temperature for 5 min. Add 2 × SDS loading buffer and boil the sample at 96 °C for 10 min.

### Binding determination

The interactions between RNF8, OPTN, and KDM6A were determined using ProteinA biosensors in the Octet R2 system (SARTORIUS Inc., Germany). First, the 293T cell lysate was extracted. The biosensors were loaded with RNF8, OPTN, and KDM6A, respectively (10 μg/ml) for 300 s. The loaded biosensors were sealed with IgG (50 μg/ml) for 600 s. Diluted 293T cell lysate in PBS solution containing 0.05% Tween-20 was then added onto the ProteinA biosensors loaded with RNF8, OPTN, or KDM6A. The real-time binding responses (Δλ in nanometers, nm) between RNF8, OPTN, and KDM6A were calculated compared with IgG and ProteinA biosensors for nonspecific binding. The kinetic parameters and affinities were calculated with a non-linear global fit of the data, using Octet data analysis software version 13.0 (SARTORIUS Inc., Germany).

### ELISA

According to the experimental steps of the human IL6 ELISA kit (Xinbosheng, EHC007.96) and the human IL1β ELISA kit (Xinbosheng, EHC002b. 96), the semen supernatant samples of 5–6 healthy subjects and oligozoospermia patients were tested and statistically analyzed.

### qPCR

Total RNA was extracted from testicular tissue and 293T cells using TRIZOL (Ambient, 95141801), and RNA concentration and purity were detected using nanodrop spectrophotometry (Thermo Scientific). cDNA synthesis was performed using the FastKing RT kit (Tiangen Biotech Co., Ltd, KR118). qPCR was performed using SYBR Green PCR Material Mixture reagent (Tiangen Biotech Co., Ltd, FP205) on the Real-time PCR Detection System (Bio-Rad), with primer sequences shown in Table S1.

### Antibodies

The following antibodies were used: RNF8 (Proteintech, #14112-1-AP), β-actin (Affinity, #AF7018), Tri-Methyl-Histone H3 (Lys27) (Affinity, #DF6941), IL6 (Affinity, #DF6087), IFN-γ (Affinity, #DF6045), γ-H2AX (Servicebio, # GB111841-100), CD86 (Affinity, #DF6332), CD74 (Immunoway, #YT5464), CD163 (Proteintech, #16646-1-AP), OPTN (Proteintech, #10837-1-AP), CD206 (Affinity, #DF4149), CD80 (Proteintech, #66406-1-Ig), p62 (Proteintech, #31403-1-AP), LC3(Proteintech, #14600-1-AP), c-kit(Affinity, #AF6153), Scp3(Proteintech, #23024-1-AP), Flag (Proteintech, #20543-1-AP), HA (Proteintech, #66006-2-Ig), His (Proteintech, #66005-1-Ig), ubiquitin (Cell Signaling Technology, #91112S), K48 (Cell Signaling Technology, #12805), K63 (Cell Signaling Technology, #5621S).

### Statistical methods

All experimental data are from three independent replicates, which are expressed as $\bar{x}\pm {\rm{SD}}$. The differences between groups were analyzed by Student's *t* test, and *p* < 0.05 was considered as statistically significant. The GraphPad Prism 9 was used for statistical analysis and drawing. *p < 0.05, ** *p* < 0.01, *** *p* < 0.001 and **** *p* < 0.0001 were considered statistically significant.

## Supplementary information

Supplementary Materials

Original Data

## Data Availability

All relevant data can be obtained from the corresponding author upon request.
