# Supplementary material for: The RNF8/OPTN/KDM6A axis controls macrophage polarization to maintain testicular microenvironment homeostasis
Source: Cell Death Discov. 2025 Jul 24;11:339. doi: 10.1038/s41420-025-02641-3 (PMC12289966; doi:10.1038/s41420-025-02641-3)
Supplement: Supplementary file 1 — Supplementary Materials [file 41420_2025_2641_MOESM1_ESM.pdf]

# Supplementary materials

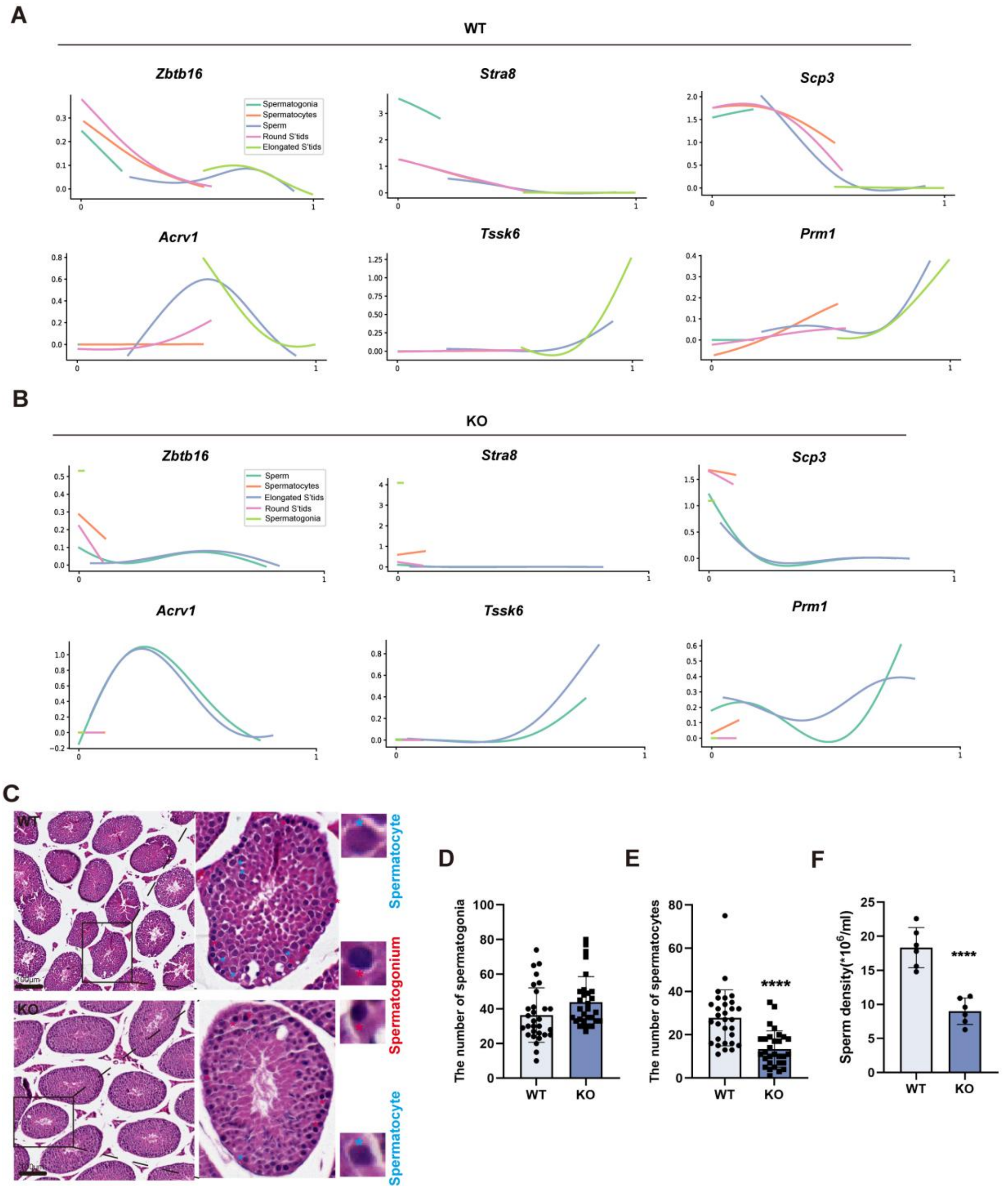


**Sup. 1 Inhibition of the spermatogenesis process in the microenvironment of RNF8$^{-/-}$ mice testes.**

A-B. Pseudotime analysis of the expression of biomarkers at different stages of germ cells in WT and KO mice (*Zbtb16, Stra8, Scp3, Acvr1, Tssk6, Prm1*). C-E. HE staining and analysis of testicular tissue in WT and KO mice, with red asterisks representing spermatogonia and blue asterisks representing spermatocytes. Scale bar 100μm. The statistics of spermatogonia (D) and spermatocytes (E) in each seminiferous tubule, n=30. F. Sperm density changes in WT and KO mice. n = 6 per group. Student's *t*-test; ****$P < 0.0001$.

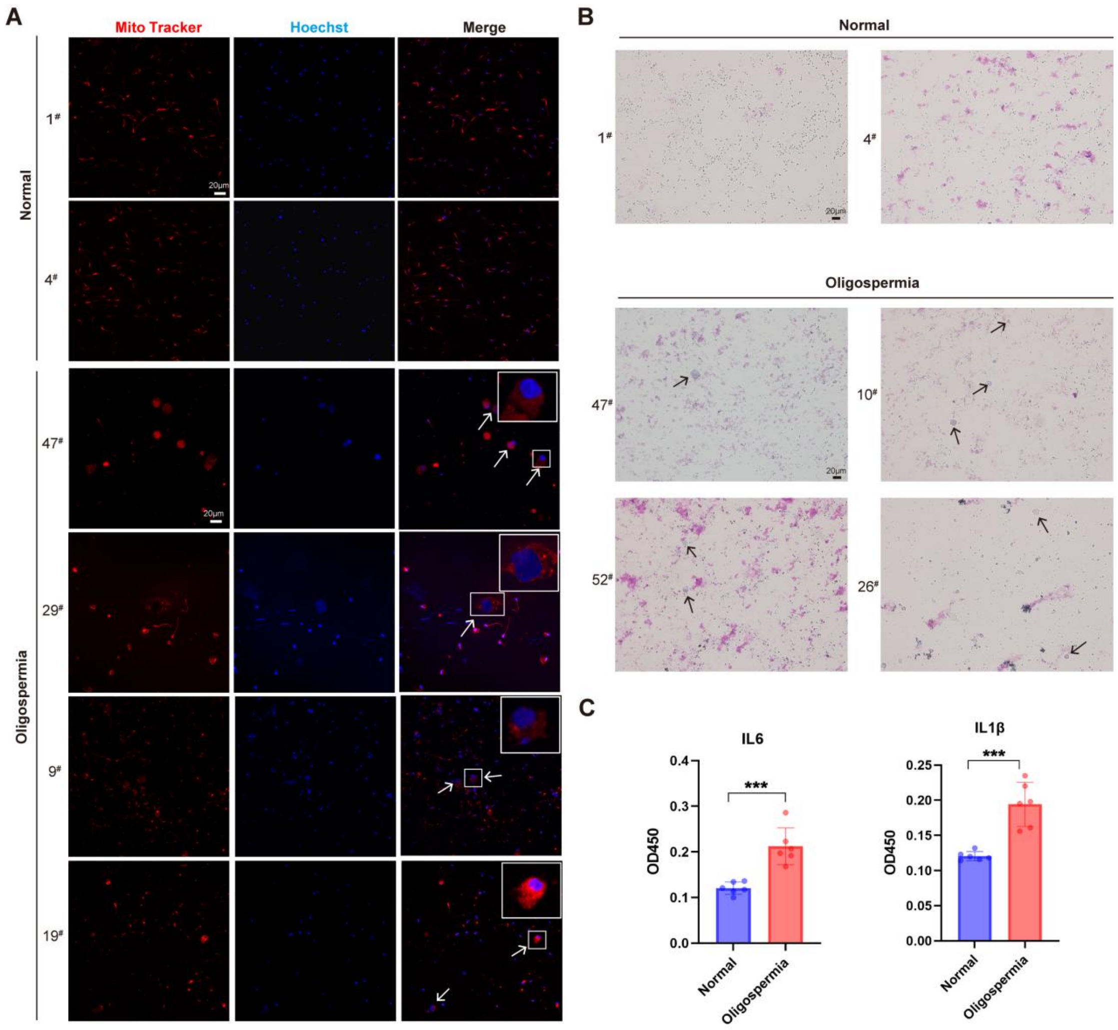


**Sup. 2 Detection of immune status in clinical semen samples.**

A. The sperm suspension of the control group and oligospermia patients was traced by fluorescence staining using Hoechst and Mitotracker, with white arrows indicating mononuclear macrophages. Scale bar 20μm. B. Giemsa staining was performed on the sperm smears of the control group and oligospermia patients, with black arrows indicating mononuclear macrophages. Scale bar 20μm. C. The difference of IL-6 and IL-1β levels in seminal plasma between control group and oligospermia patients was detected by ELISA, n=6. All values were presented as the mean ± SD. Student's *t*-test; ***$P < 0.001$.

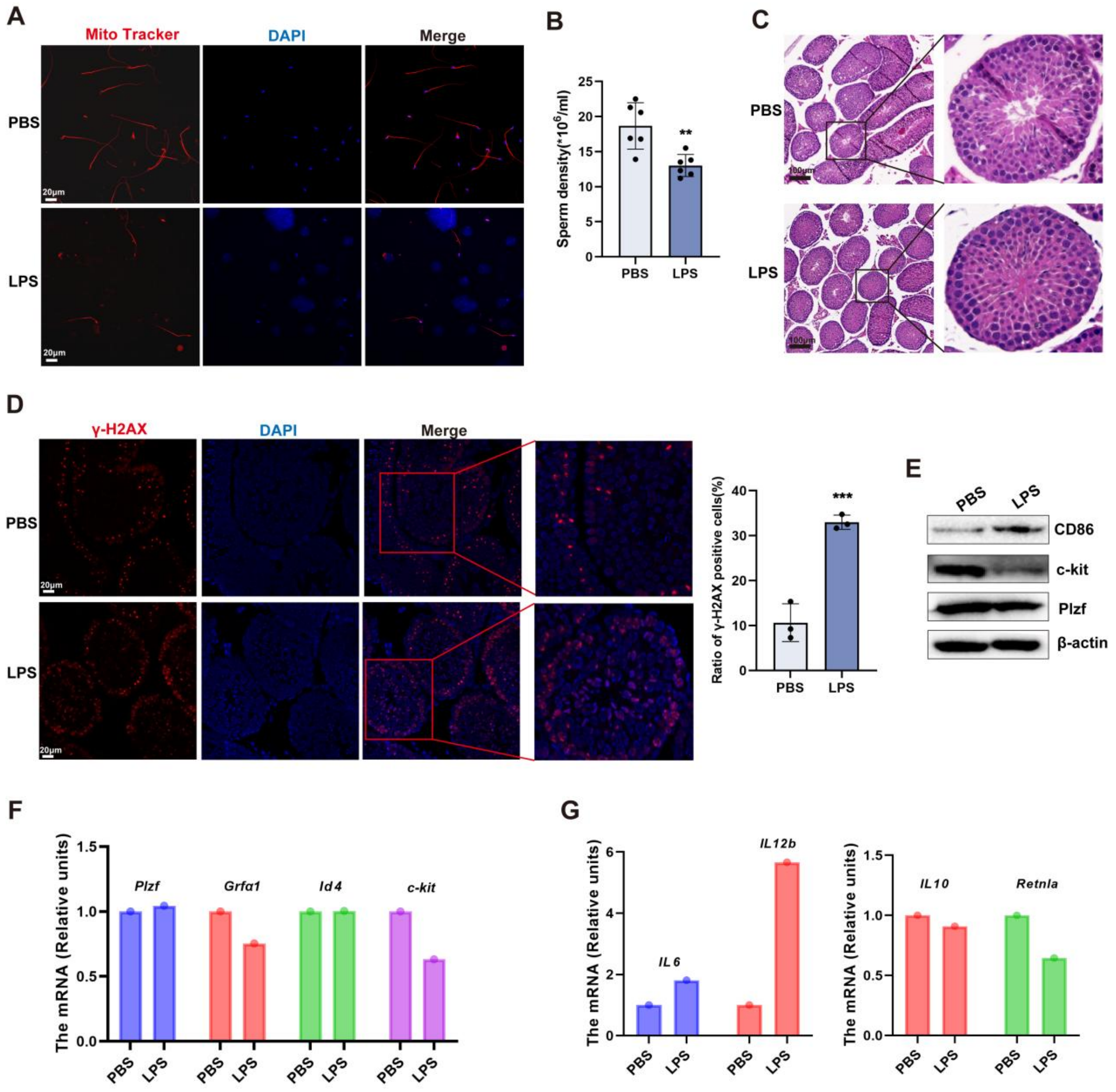


**Sup. 3 LPS-induced systemic immune response impaired differentiation of testicular spermatogenic cells.**

A. Mitochondrial staining of sperm in the control group (PBS) and LPS intraperitoneal injection group (1.5 mg/kg). Scale bar 20μm. B. Sperm density changes in the control group and LPS intraperitoneal injection group. n = 6 per group. C. HE staining of testicles in the control group and LPS intraperitoneal injection group. Scale bar 100μm. D. Immunofluorescence staining and analysis of testicular γ-H2AX in the control group and LPS intraperitoneal injection group. Scale bar 20μm. E. The western blot of CD86, c-kit and Plzf in the control group and LPS intraperitoneal injection group. F. The mRNA of sperm development (Plzf, Grfα1, Id4, c-kit) in the control group and LPS intraperitoneal injection group. G. The mRNA of pro-inflammatory macrophages (IL6, IL12b) and anti-inflammatory macrophage (IL10, Retnla) marker in the control group and LPS intraperitoneal injection group. Student's t-test; $^{**}P < 0.01$, $^{***}P < 0.001$.

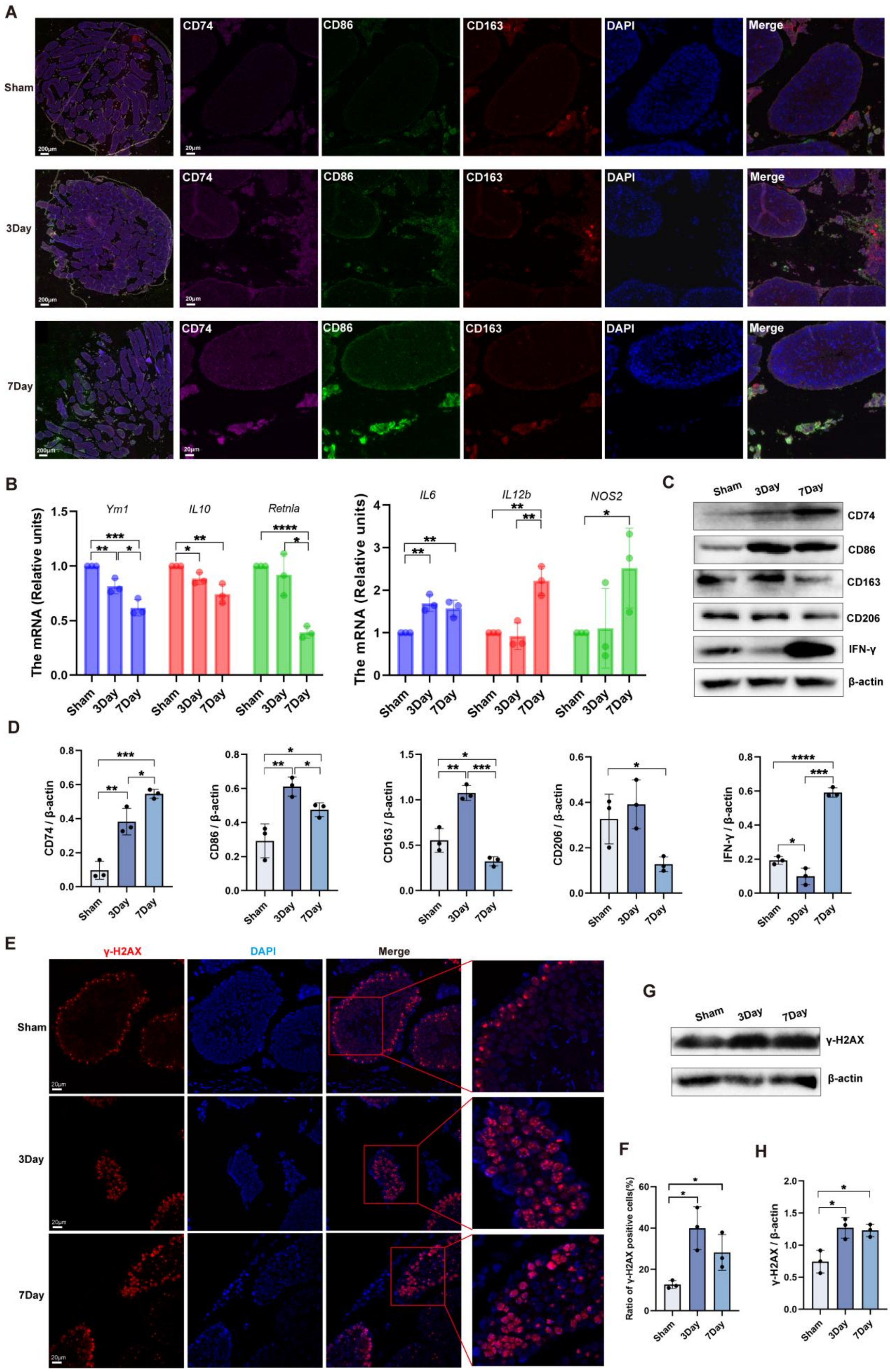


**Sup. 4 Macrophage pro-inflammatory polarization led to enhanced inflammation in testicular microenvironment and increased damage in spermatogenic cells.**

A. The multiple immunofluorescences staining of CD74, CD86, and CD163 in sham group and pro-Mφ injection for 3 days and 7 days group. Scale bar is 200μm and 20μm respectively. B. The mRNA of *Ym1*, *IL10*, *Retnla*, *IL6*, *IL12b* and *NOS2* in testis were detected in the sham group and pro-Mφ injection group. C-D. The western blot and analysis of CD74, CD86, CD163, CD206 and IFN-γ in testis were detected in the sham group and pro-Mφ injection group. E-F. The immunofluorescence and analysis of γ-H2AX in the testis of sham group and pro-Mφ injection group. Scale bar 20μm. G-H. The western blot and analysis of γ-H2AX in the testis of sham group and pro-Mφ injection group. All values were presented as the mean ± SD. Student's *t*-test; $*P < 0.05$, $**P < 0.01$, $***P < 0.001$, $****P < 0.0001$.

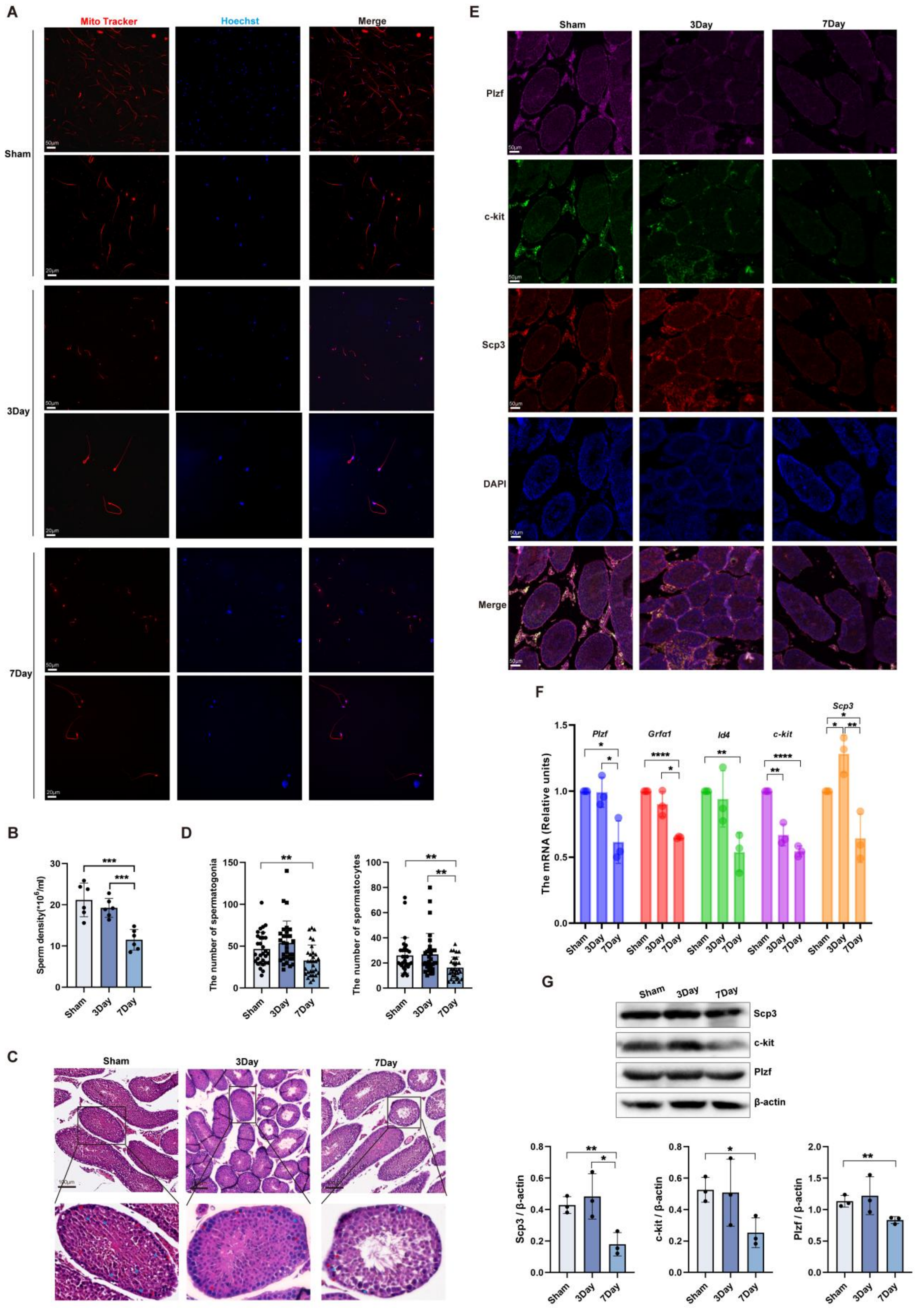


**Sup. 5 Pro-inflammatory polarization of macrophages led to exhaustion in the differentiation of testicular spermatogenic cells.**

A. Staining of sperm mitochondria in the epididymis of the sham group and pro-Mφ injection

group. Scale bar is 50μm and 20μm respectively. B. Sperm density changes in sham group and pro-Mφ injection group. n = 6 per group. C-D. HE staining (red asterisk indicating spermatogonia, blue asterisk indicating spermatocytes) and statistics of spermatogonia and spermatocytes in each seminiferous tubule of the control group and pro-Mφ injection group, n=30. Scale bar 100μm. E. The multiple immunofluorescences staining of Plzf, c-Kit, and scp3 in the sham group and pro-Mφ injection group. Scale bar 50μm. F. The mRNA of *Plzf*, *Grfα1*, *Id4*, *c-kit* and *Scp3* in the sham group and the pro-Mφ injection group. G. The protein of Scp3, Plzf and c-kit in the sham group and the pro-Mφ injection group. All values were presented as the mean ± SD. Student's *t*-test; *$P < 0.05$, **$P < 0.01$, ***$P < 0.001$, ****$P < 0.0001$.

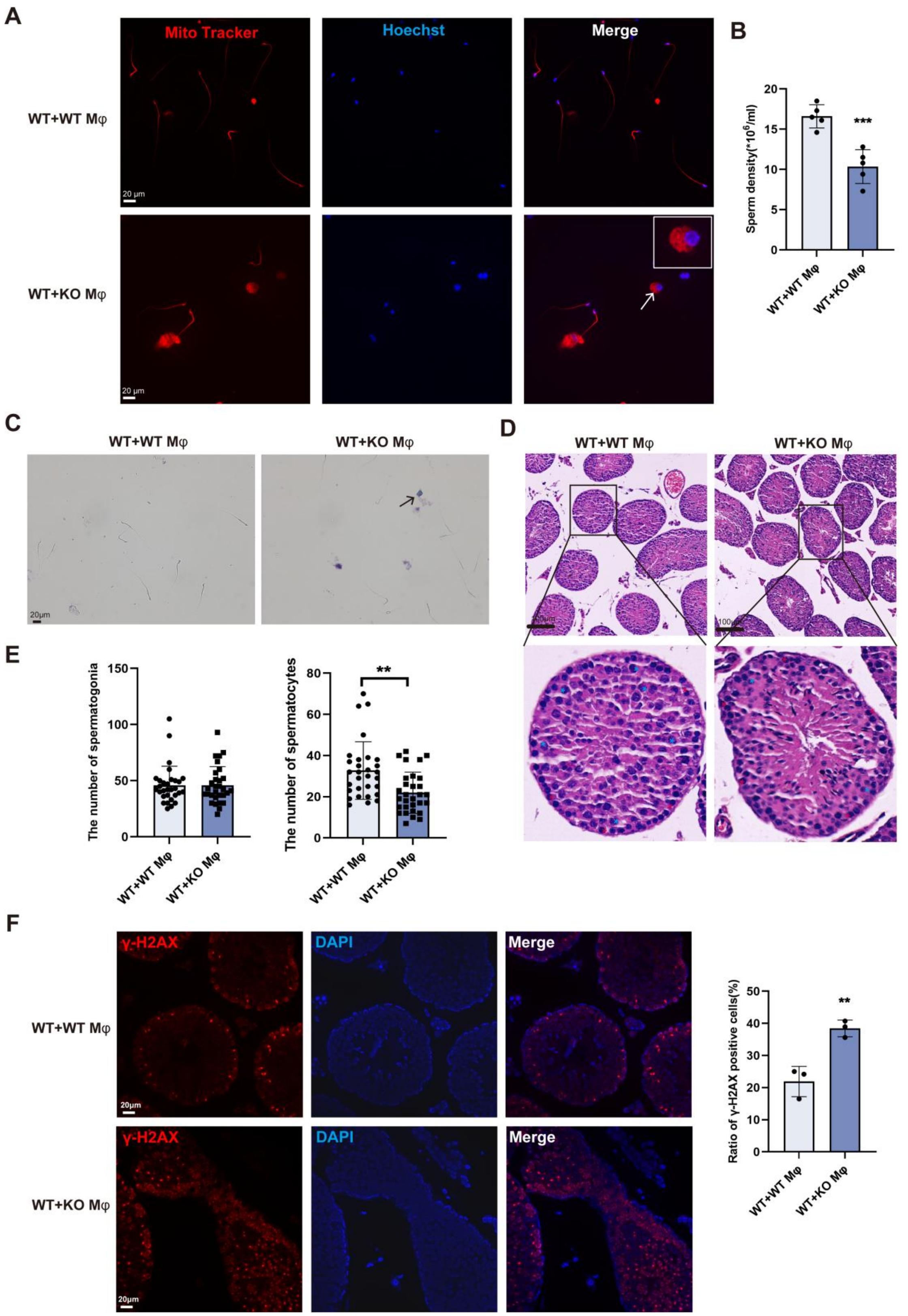


**Sup. 6 RNF8 deficiency mice peritoneal macrophages promoted intratesticular inflammatory response.**

A. Sperm suspensions of wild-type mice injected with WT and KO peritoneal macrophages were

traced using Hoechst and mitotracker staining, with white arrows indicating mononuclear macrophages. Scale bar 20μm. B. Sperm density changes in WT+WT Mφ group and WT+KO Mφ group. n = 5 per group. C. Giemsa staining was performed on the sperm smears of the above two groups, and the black arrows indicated mononuclear macrophages. Scale bar 20μm. D-E. HE staining of testis tissue of two groups of mice, the red asterisk indicates spermatogonia, the blue asterisk indicates spermatocytes, and the statistics of spermatogonia and spermatocytes in each seminiferous tubule, n=30. Scale bar 100μm. F. Immunofluorescence staining was used to detect the expression of γ-H2AX in the testis of the above two groups of mice. Scale bar 20μm. All values were presented as the mean ± SD. Student's *t*-test; $^{**}P < 0.01$, $^{***}P < 0.001$.

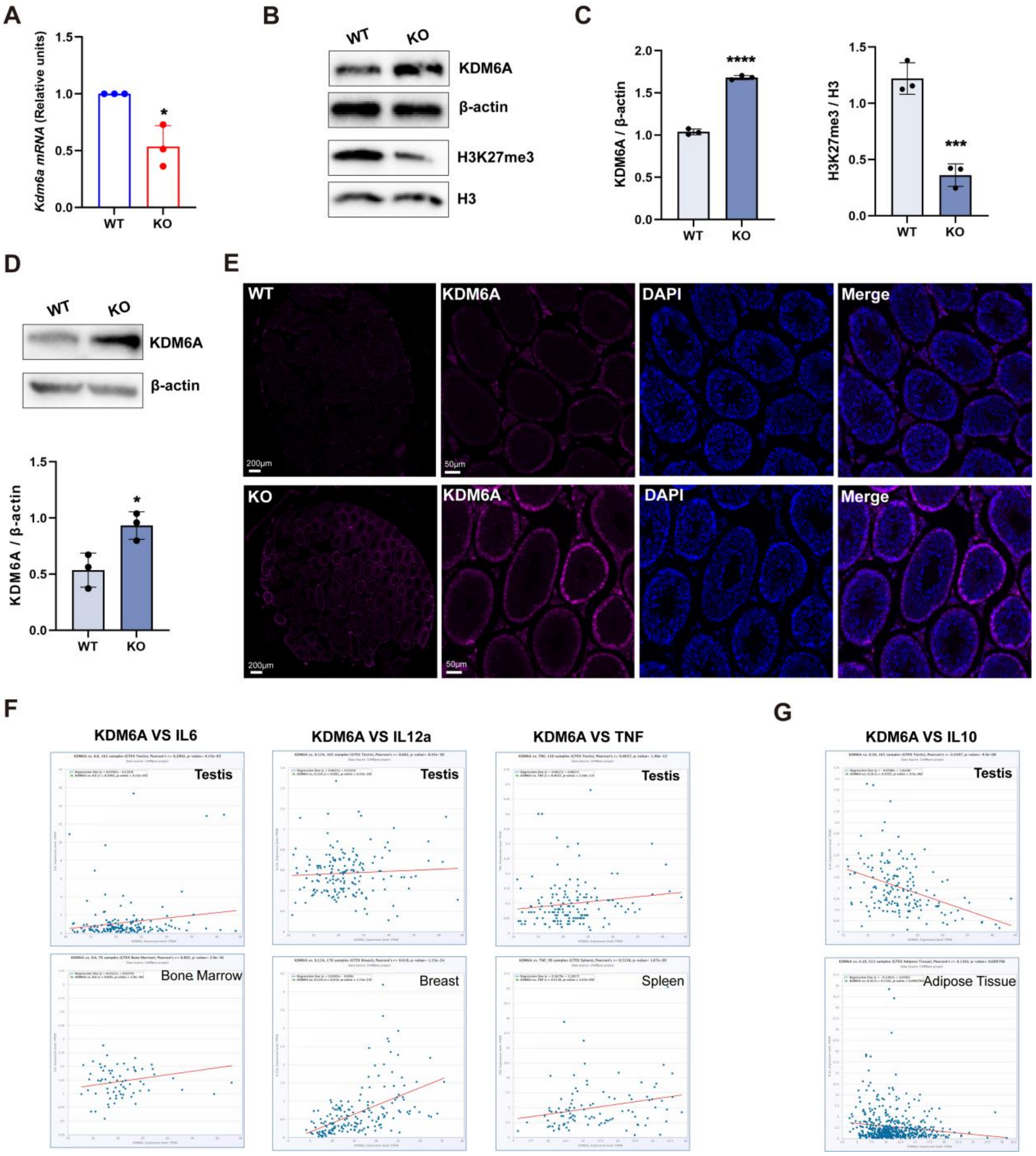


**Sup. 7 RNF8 affected macrophage pro-inflammatory polarization by regulating KDM6A.**

A. The qPCR was used to detect the mRNA expression levels of *KDM6A* in the peritoneal macrophages of WT and KO mice. B-C. Western blot was used to detect the protein levels of KDM6A and H3K27me3 in the peritoneal macrophages of WT and KO mice. D. Western blot was used to detect the expression of KDM6A protein in the testicular tissues of WT and KO mice. E. Immunofluorescence staining was used to detect the distribution of KDM6A in the testicular tissues of WT and KO mice. Scale bar is 200μm and 50μm respectively. F. Analyze the correlation between KDM6A and IL6, IL12a, and TNF in the GTEx database. G. Analyze the correlation between KDM6A and IL10 in the GTEx database. All values were presented as the mean ± SD.

Student's $t$-test; $*P < 0.05$, $***P < 0.001$, $****P < 0.0001$.

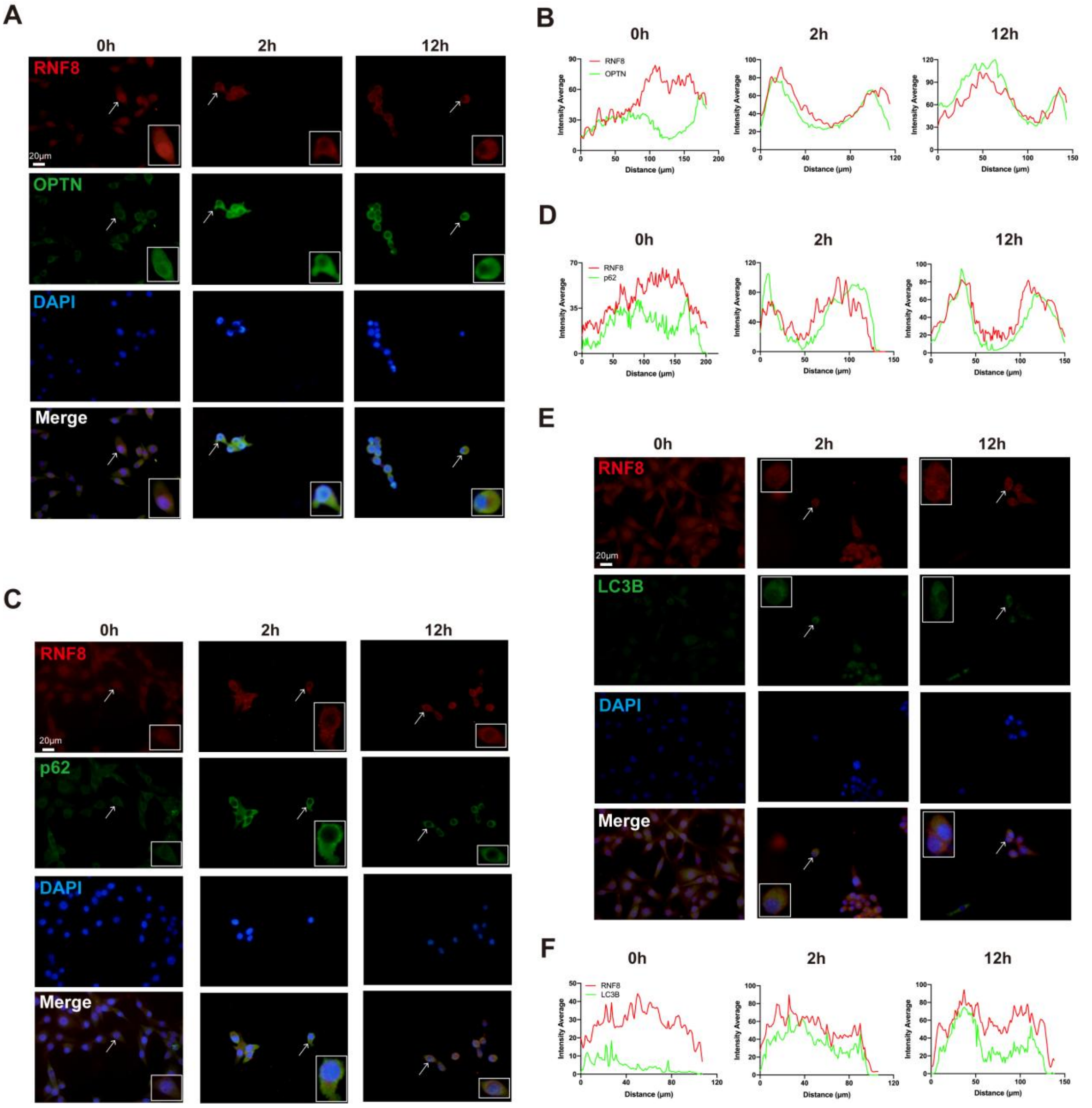


**Sup. 8 Autophagy promoted co-expression of RNF8 and its related genes.**

A-B. EBSS cultured 293T cells for 0, 2, and 12 h, and immunofluorescence was used to detect the co-localization of RNF8 with OPTN. C-D. The co-localization of RNF8 with p62. E-F. The co-localization of RNF8 with LC3B (LC3II). Scale bar 20µm.

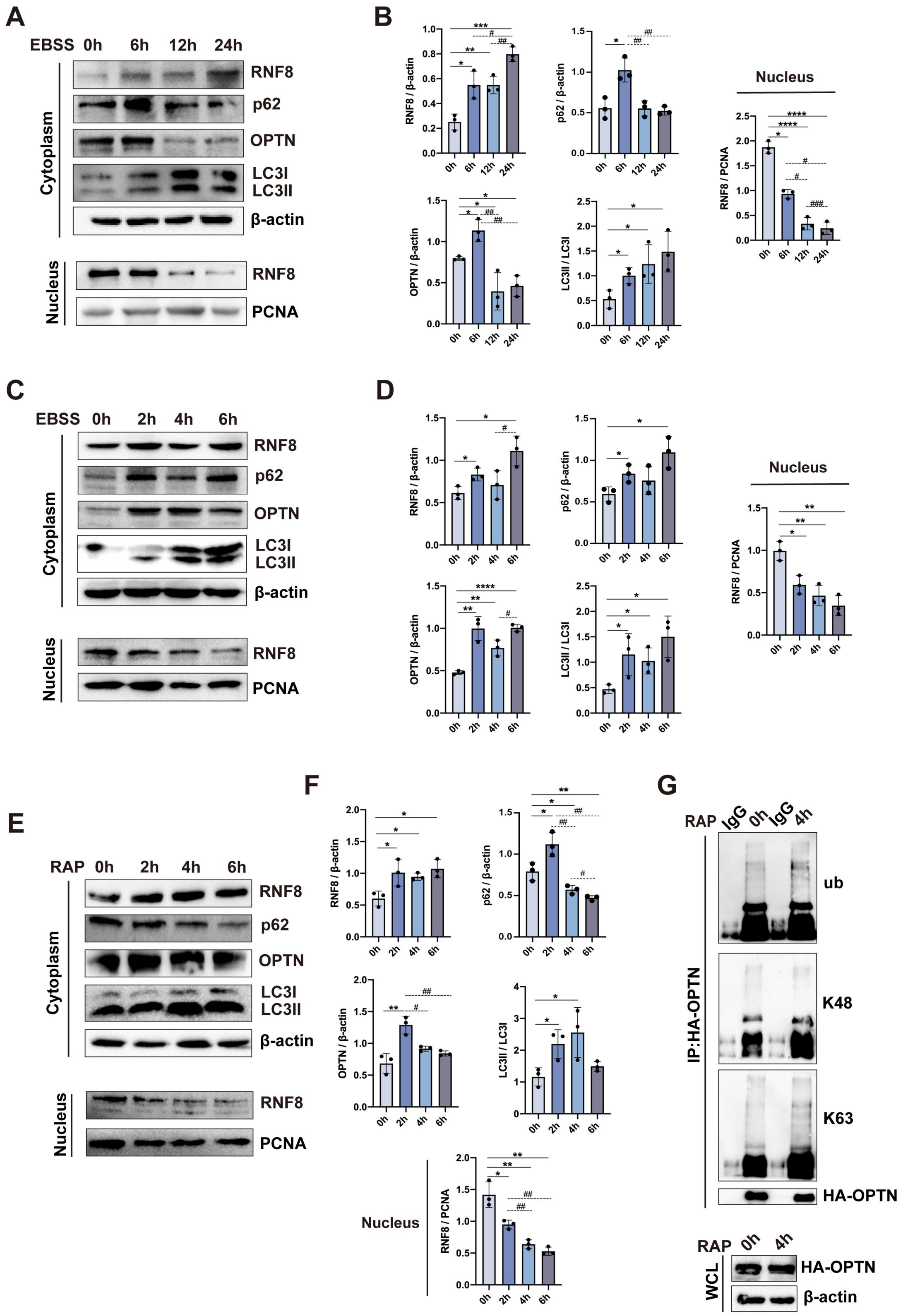


**Sup. 9 The expression of RNF8 in cytoplasm promoted autophagy progression.**

A-B. EBSS induced 293T cells to detect and analyze the expression of RNF8 in the cytoplasm and nucleus, and the expression of p62, OPTN and LC3 in the cytoplasm at 0h, 6h, 12h, and 24h respectively. C-D. EBSS induced 293T cells to detect at 0h, 2h, 4h, and 6h respectively, then

detected and analyzed the expression of RNF8, p62, OPTN and LC3. E-F. RAP induced 293T cells for 0 h, 2 h, 4 h, and 6 h respectively, and the above indexes were detected and analyzed. G. After 4h of RAP induction, changes in ubiquitin, K48-linked ubiquitination and K63-linked ubiquitination levels were detected by IP-HA (OPTN). All values were presented as the mean ± SD. Student's *t*-test; *$P < 0.05$, **$P < 0.01$, ***$P < 0.001$, ****$P < 0.0001$.

**Table S1. Primer sequence of real-time PCR**

| Primer | Forward Primer sequences(5'→3') | Reverse Primer sequences(5'→3') |
|---|---|---|
| Kdm6a | CGGGCGGACAAAAGAAGAAC | CATAGACTTGCATCAGATCCTCC |
| Nos2 | ACATCGACCCGTCCACAGTAT | CAGAGGGGTAGGCTTGTCTC |
| IL6 | CCACGGCCTTCCCTACTTC | TTGGGAGTGGTATCCTCTGTGA |
| Arg1 | CTCCAAGCCAAAGTCCTTAGAG | GGAGCTGTCATTAGGGACATCA |
| Retnla | CCAATCCAGCTAACTATCCCTCC | ACCCAGTAGCAGTCATCCCA |
| Ym1 | ACTGATAGCAGTTTGCCCAAG | TCTACGTTCCCCAAGTCGTTAG |
| IL10 | ACACTGCCTGAGACCTTGTTG | GGAGCCCGTCCTCTTGCTA |
| IL12b | TTGAACTGGCGTTGGAAGCACG | CCACCTGTGAGTTCTTCAAAGGC |
| Plzf | CTGGGACTTTGTGCGATGTG | CGGTGGAAGAGGATCTCAAACA |
| Grfα1 | TGCGTATCTACTGGAGCATGT | CATCGAGGCAGTTGTTCCCTT |
| Id4 | CAGTGCGATATGAACGACTGC | GACTTTCTTGTTGGGCGGGAT |
| C-kit | GGCCTCACGAGTTCTATTTACG | GGGGAGAGATTTCCCATCACAC |
| Scp3 | AGCCAGTAACCAGAAAATTGAGC | CCACTGCTGCAACACATTCATA |

**Table S2. Anti-sperm antibody for healthy individuals and patients with oligozoospermia**

| Age | AsAb-IgA | AsAb-IgG | AsAb-IgM |
|---|---|---|---|
| 40 | (-) | (-) | (-) |
| 37 | (-) | (-) | (-) |
| 28 | (-) | (-) | (-) |
| 31 | (-) | (-) | (-) |
| 29 | (-) | (-) | (-) |
| 30 | (-) | (-) | (-) |
| 38 | (-) | (-) | (-) |
| 42 | (-) | (-) | (-) |
| 28 | (-) | (-) | (-) |
| 41 | (-) | (-) | (-) |

**Table S3. Urinalysis for healthy individuals and patients with oligozoospermia**

| Age | PH | Occult blood test | Urine protein | Urobilinogen | Nitrite | Hemameba | Bacteria (PCS /uL) | Fungi (PCS /uL) |
|---|---|---|---|---|---|---|---|---|
| 37 | 5. 0 | (±) | (-) | (-) | (-) | (-) | 0 | 0 |
| 28 | 5. 0 | (-) | (-) | (-) | (-) | (-) | 0 | 0 |
| 33 | 5. 0 | (-) | (-) | (-) | (-) | (-) | 0 | 0 |
| 31 | 6. 0 | (-) | (-) | (-) | (-) | (-) | 0 | 0 |
| 29 | 5. 0 | (-) | (±) | (-) | (-) | (-) | 0 | 0 |
| 38 | 6. 0 | (-) | (+) | (-) | (-) | (-) | 0 | 0 |
| 42 | 6. 0 | (-) | (-) | (-) | (-) | (-) | 0 | 0 |
| 41 | 5. 0 | (-) | (-) | (-) | (-) | (-) | 0 | 0 |

**Table S4. Routine blood test for healthy individuals and patients with oligozoospermia**

| Age | Hemameba ($10^9$/L) | Erythrocyte ($10^{12}$/L) | Hemoglobin (g/L) | Thrombocyte ($10^9$/L) | Lymphocyte percentage (%) | Monocyte percentage (%) | Neutrophil percentage (%) | Eosinophils percentage (%) | Basophilic granulocytes percentage (%) |
|---|---|---|---|---|---|---|---|---|---|
| 37 | 7.21 | 5.39 | 169.00 | 255 | 34.7 | 3.6 | 59.9 | 1.6 | 0.2 |
| 28 | 7.89 | 5.41 | 157 | 153 | 33.0 | 5.6 | 59.2 | 1.8 | 0.4 |
| 31 | 5.52 | 4.98 | 156 | 191 | 18.8 | 11.7 | 68.4 | 0.7 | 0.4 |
| 29 | 6.32 | 5.57 | 169 | 265 | 46.0 | 5.5 | 45.4 | 2.6 | 0.5 |
| 38 | 6.78 | 6.04 | 178 | 227 | 36.9 | 4.8 | 56.9 | 0.7 | 0.7 |
| 42 | 8.57 | 4.75 | 146 | 168 | 23.9 | 3.6 | 68.7 | 3.6 | 0.2 |
| 28 | 6.11 | 5.35 | 161 | 275 | 40.2 | 4.8 | 52.5 | 1.9 | 0.6 |
| 41 | 5.18 | 4.90 | 165 | 235 | 24.3 | 3.0 | 69.7 | 1.6 | 1.4 |

Leukocyte range 3.50-9.50 $\times 10^9$/L, erythrocyte range 4.30-5.80 $\times 10^{12}$/L, hemoglobin range 130-175g/L, platelet range 125-350 $\times 10^9$/L, lymphocyte percentage range 20.0-50.0%, monocyte percentage range 3.0-10.0%, neutrophil percentage range 40.0-75.0%, eosinophils percentage range 0.4-8.0%, Basophilic granulocytes percentage range 0-1.0%.

**Table S5. Semen information for healthy individuals and patients with oligozoospermia**

| | Healthy humans | | | | | Oligospermia patients | | | | |
|---|---|---|---|---|---|---|---|---|---|---|
| Age | 45 | 39 | 28 | 29 | 31 | 37 | 34 | 30 | 29 | 30 |
| PH | 8.4 | 7.9 | 7.9 | 7.9 | 7.9 | 7.9 | 7.9 | 7.9 | 7.9 | 7.9 |
| Sperm concentration (million/mL) | 89.4 | 66.6 | 138.7 | 90.4 | 84.1 | 10.3 | 2.4 | 6.0 | 0.8 | 2.8 |
| Total sperm number (million/ejaculate) | 259.1 | 512.7 | 721.1 | 397.8 | 462.7 | 33.0 | 9.0 | 19.4 | 1.8 | 9.0 |
| Progressive motility (%) | 61.9 | 54.6 | 46.5 | 67.1 | 60.3 | 23.0 | 16.7 | 26.4 | 11.4 | 16.2 |
| Nonprogressive motility (%) | 10.9 | 9.1 | 13.5 | 9.7 | 9.4 | 12.3 | 5.8 | 6.8 | 8.6 | 12.1 |
| Total motility (% progressive + nonprogressive ) | 72.8 | 63.7 | 60.0 | 76.8 | 69.7 | 35.3 | 22.5 | 33.2 | 20 | 28.3 |

Semen indicators of healthy individuals：Sperm concentration>15 million/ml, Total sperm number>39 million/ejaculate, Progressive motility>32%, Total motility>40%.
