# Supplementary material for: The RNF8/OPTN/KDM6A axis controls macrophage polarization to maintain testicular microenvironment homeostasis
Source: Cell Death Discov. 2025 Jul 24;11:339. doi: 10.1038/s41420-025-02641-3 (PMC12289966; doi:10.1038/s41420-025-02641-3)

Fig. 3 RNF8 deficiency promoted pro-inflammatory polarization of macrophages in the testicular microenvironment and led to spermatogenic dysfunction.

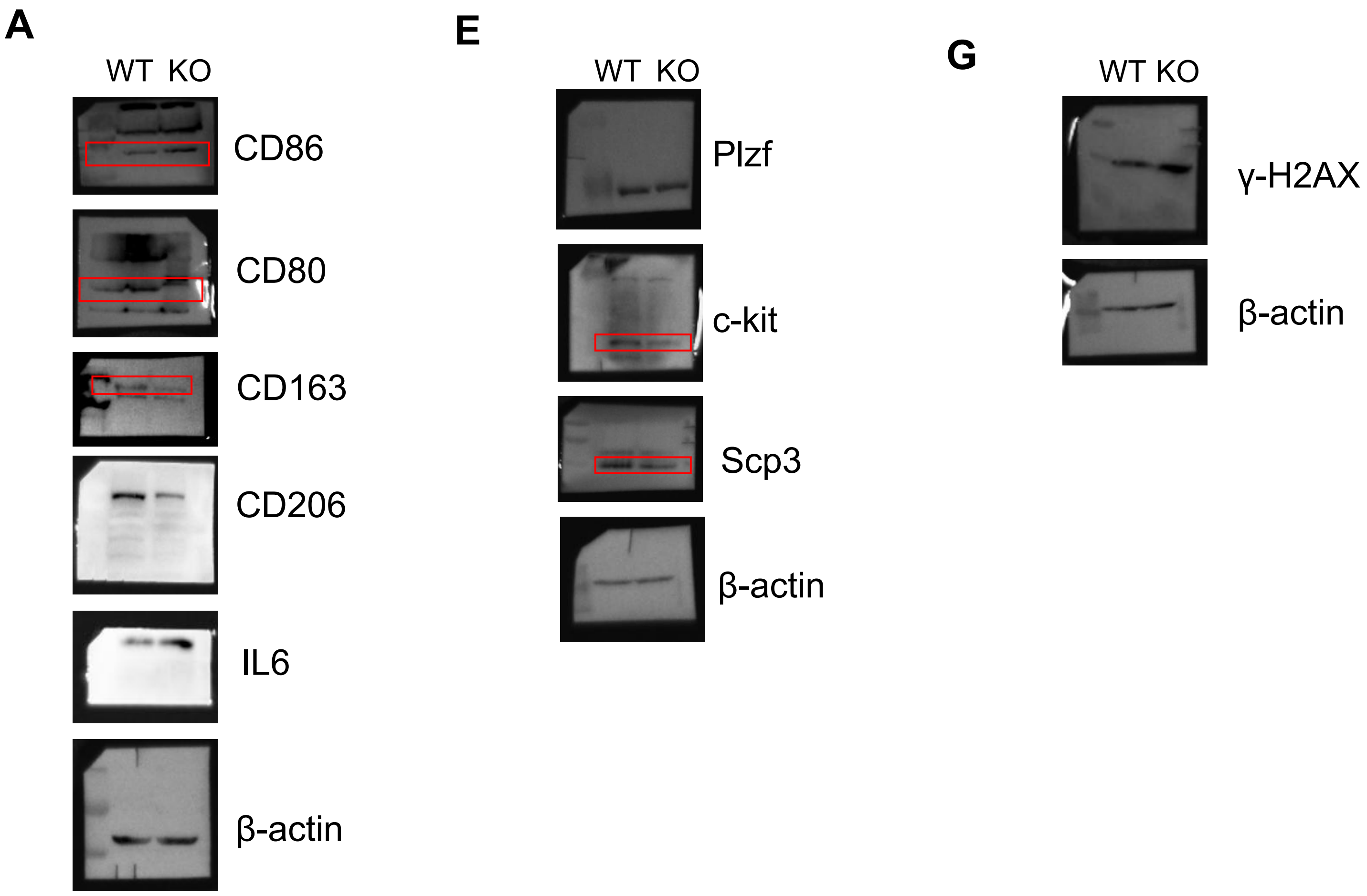

Fig. 4 RNF8$^{-/-}$ mice peritoneal macrophages hindered the development of spermatogenesis.

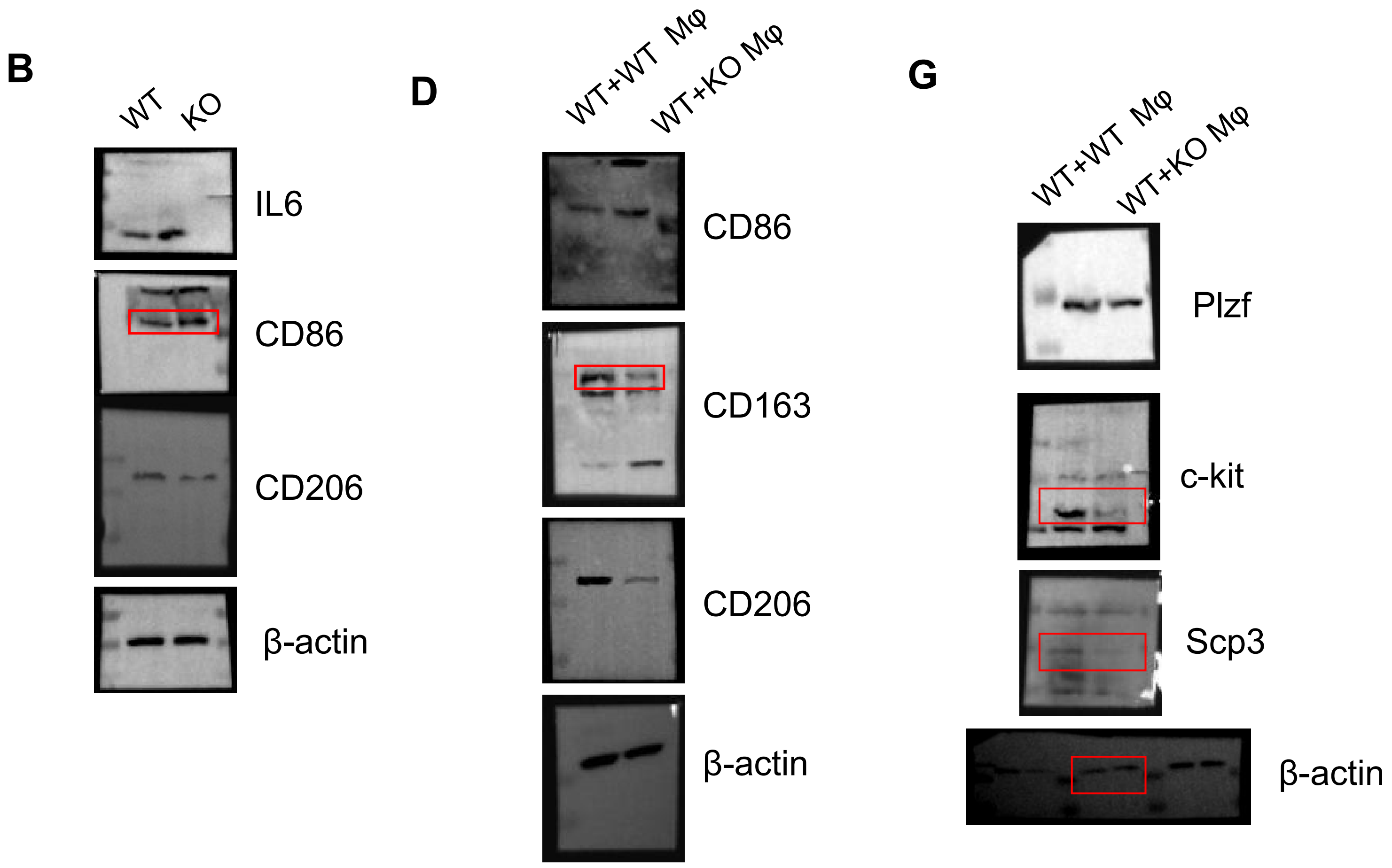

Fig. 5 RNF8 regulated the expression of H3K27me3 through KDM6A.

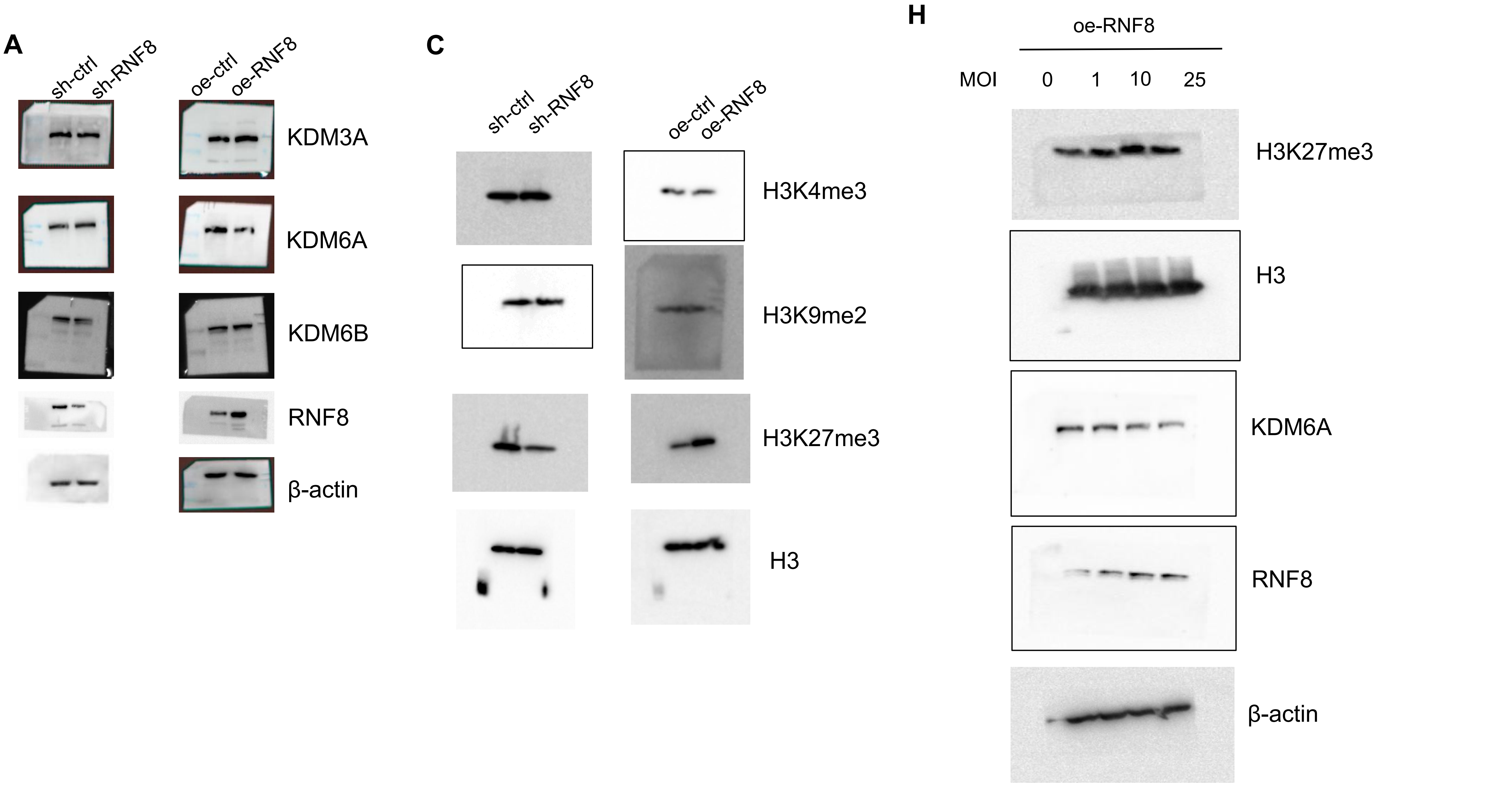

Fig. 5 RNF8 regulated the expression of H3K27me3 through KDM6A.

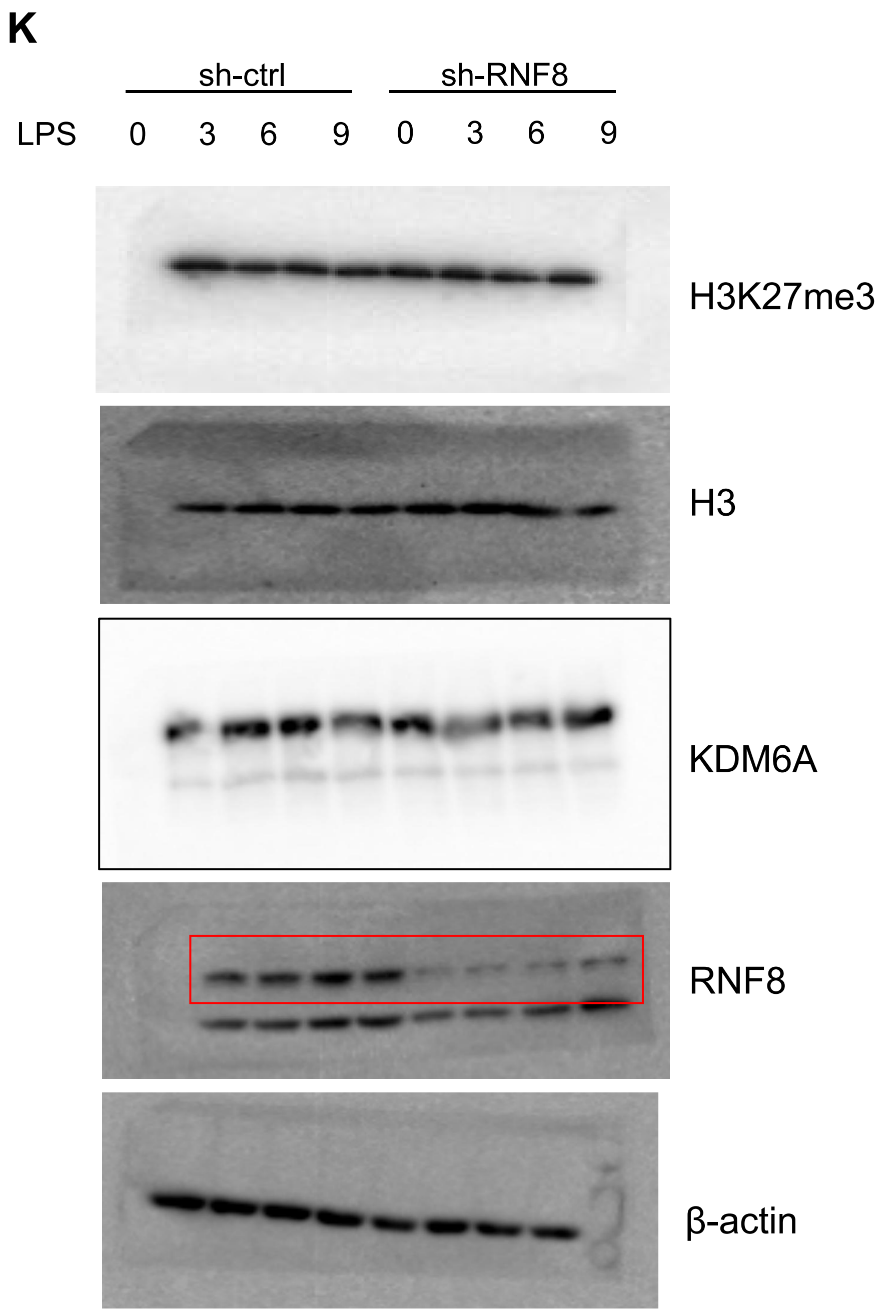

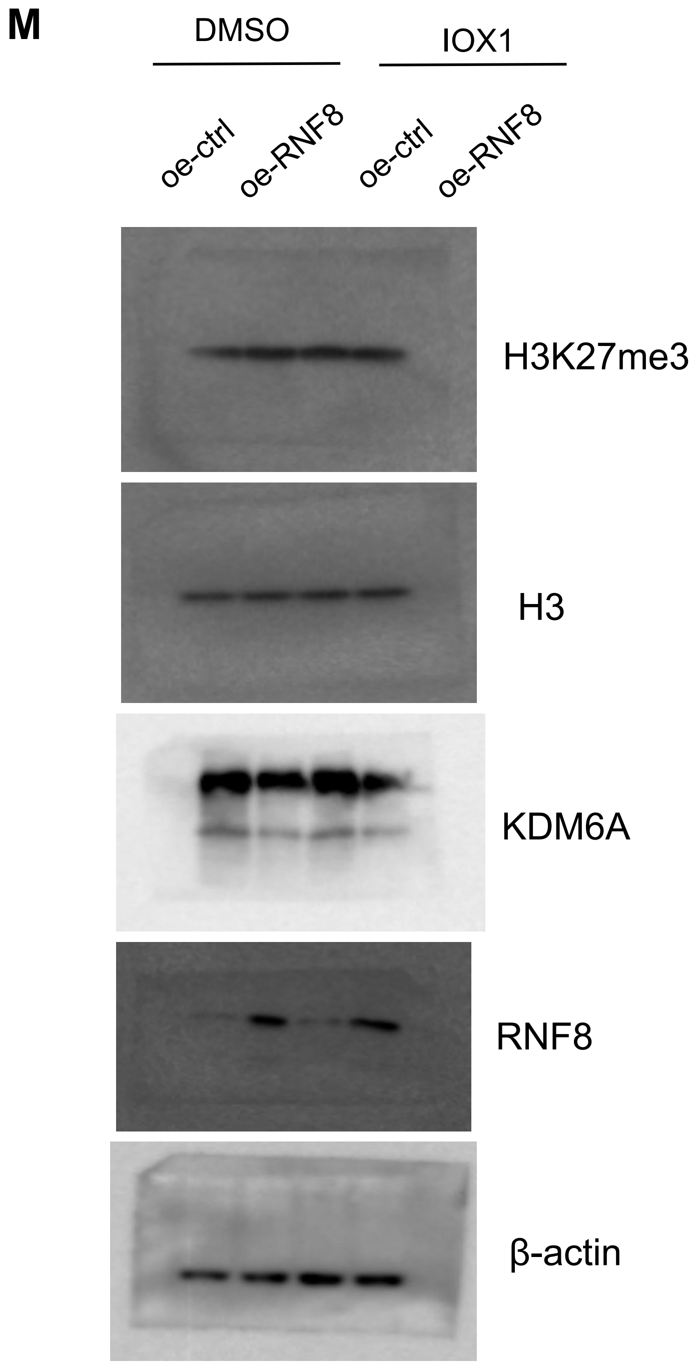

Fig. 6 RNF8 regulated the degradation of KDM6A through the autophagy-lysosome pathway.

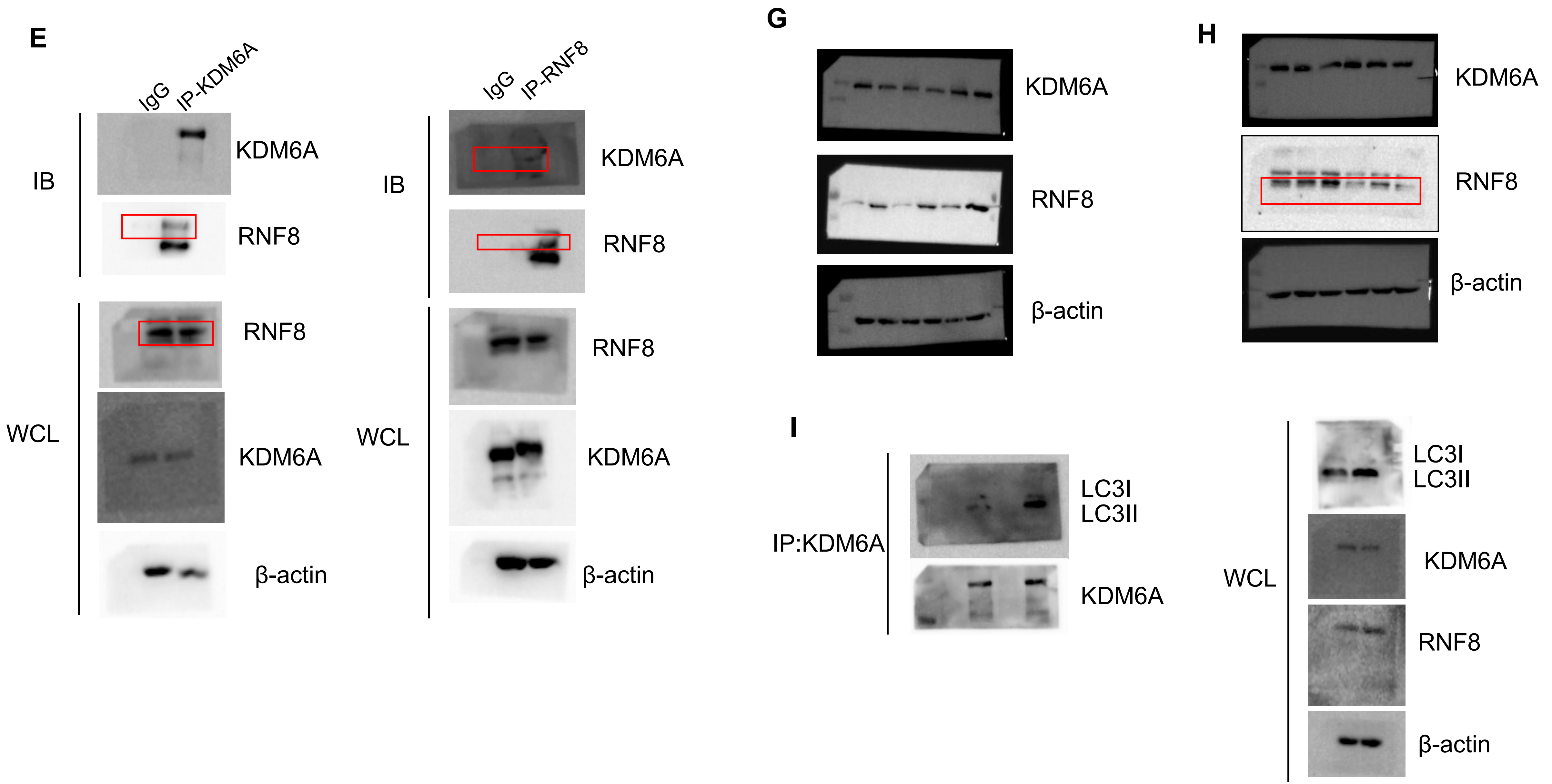

Fig. 6 RNF8 regulated the degradation of KDM6A through the autophagy-lysosome pathway.

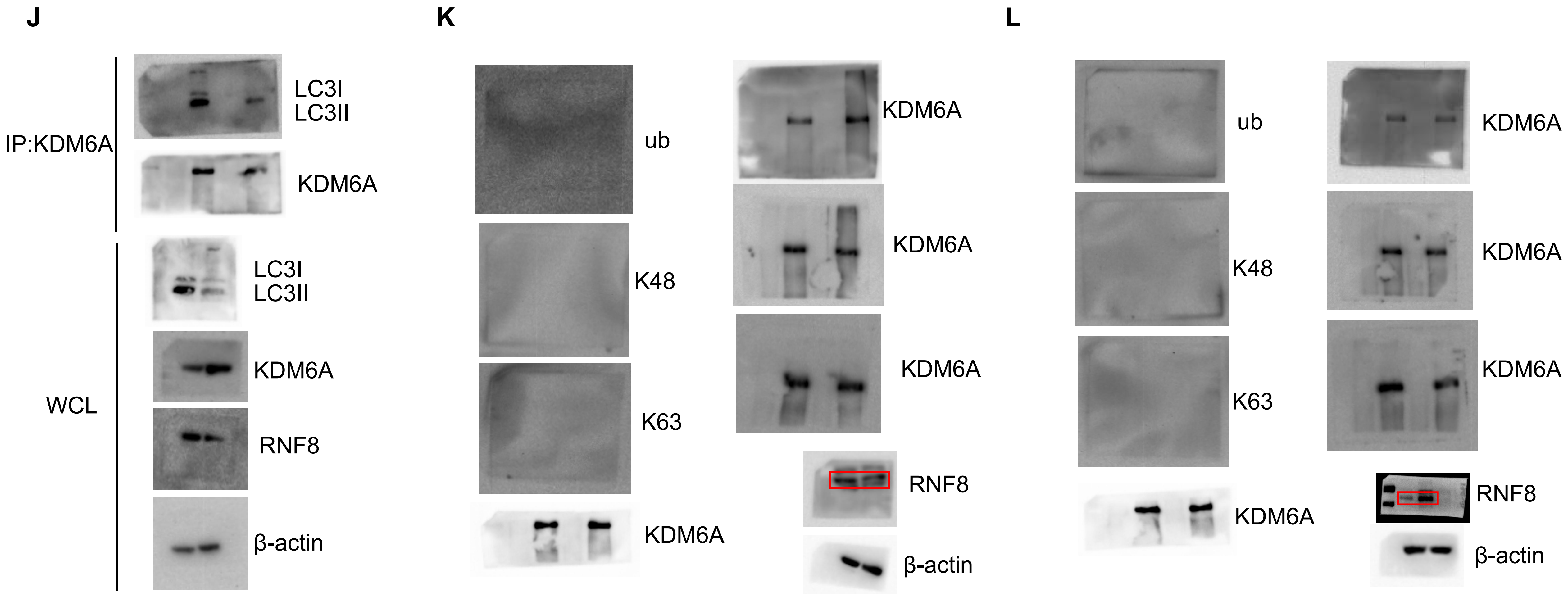

Fig. 7 RNF8 regulated the activity of OPTN by mediating K63 ubiquitination modification.

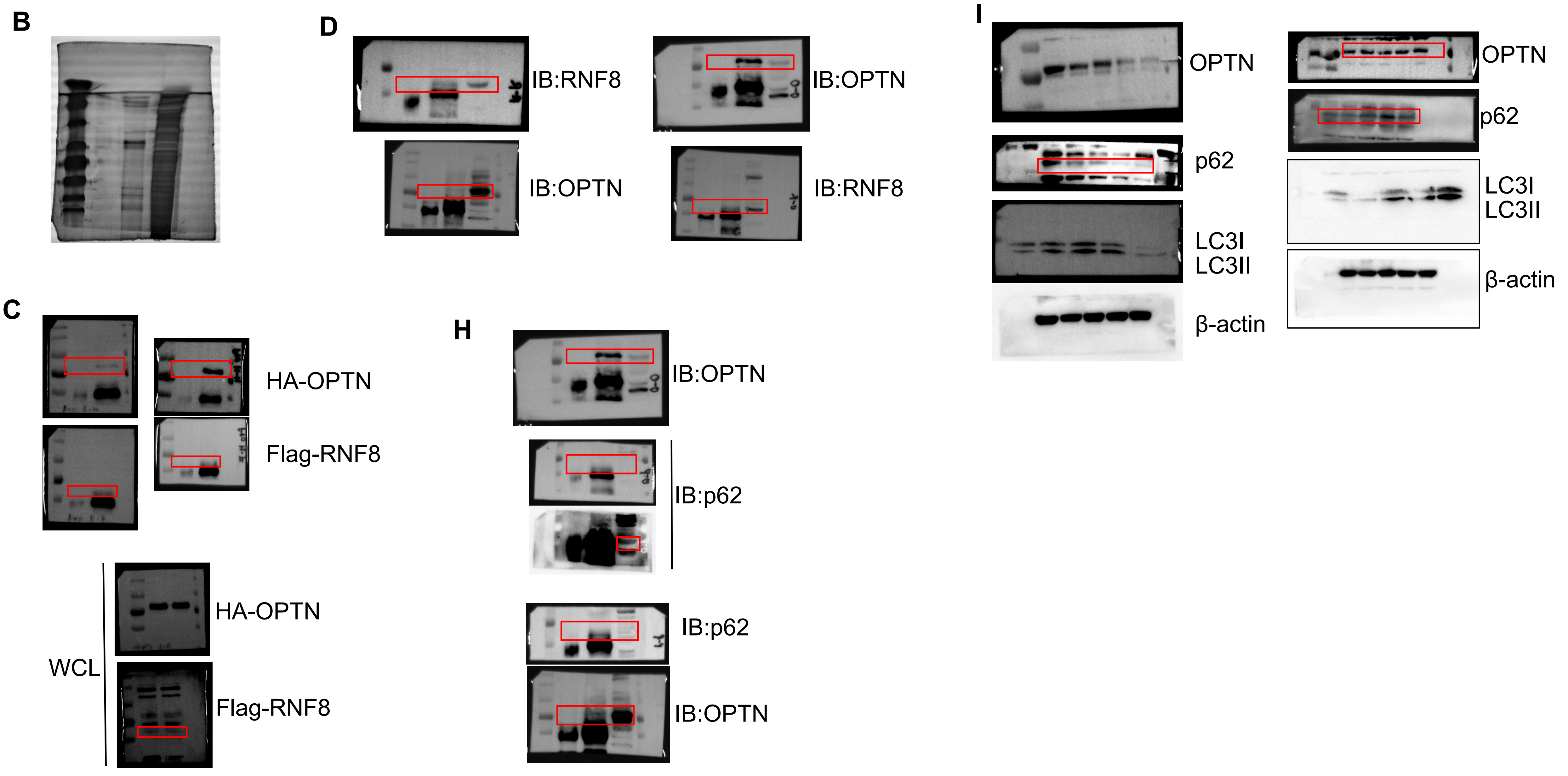

Fig. 7 RNF8 regulated the activity of OPTN by mediating K63 ubiquitination modification.

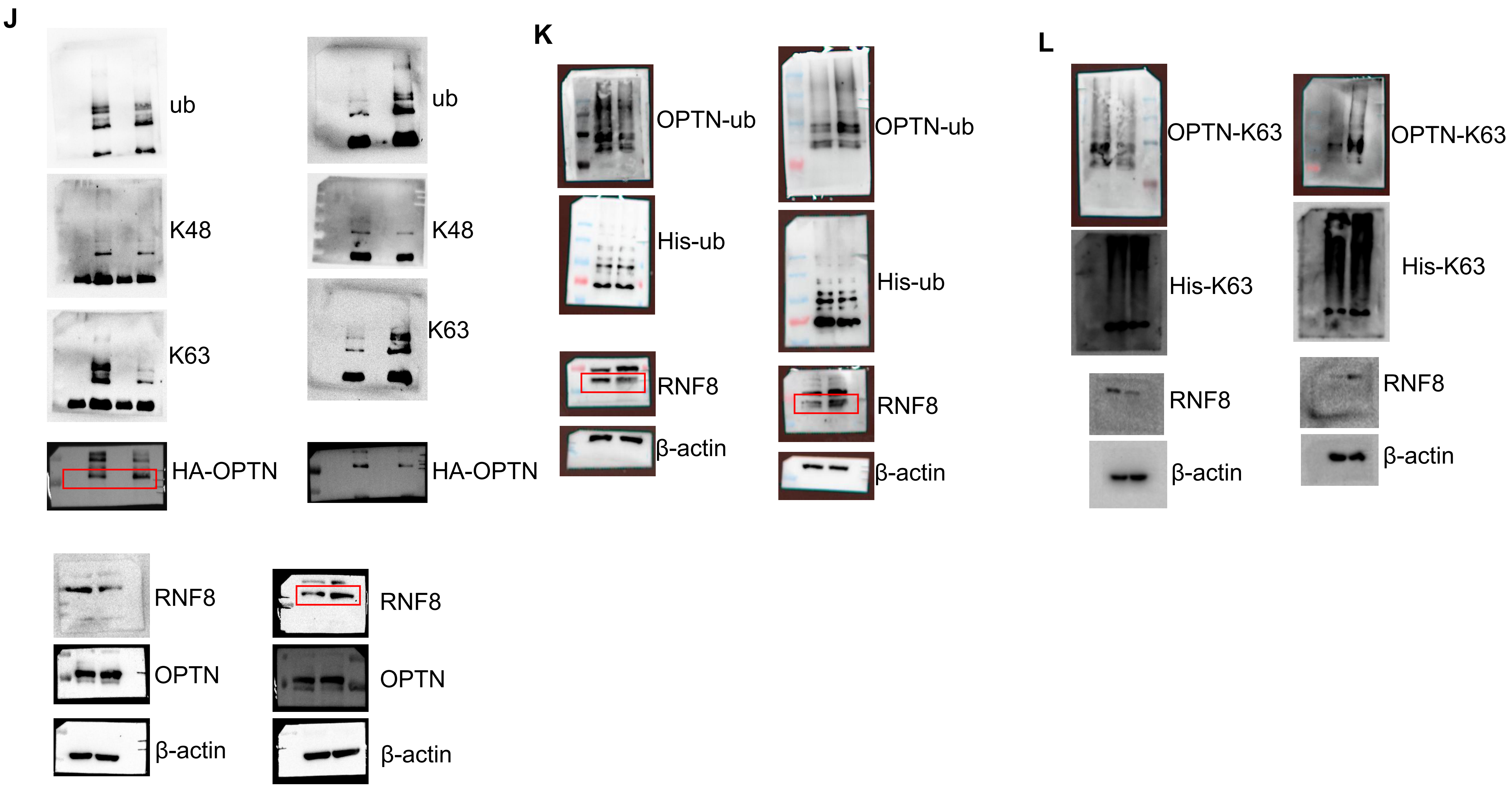

Fig. 7 RNF8 regulated the activity of OPTN by mediating K63 ubiquitination modification.

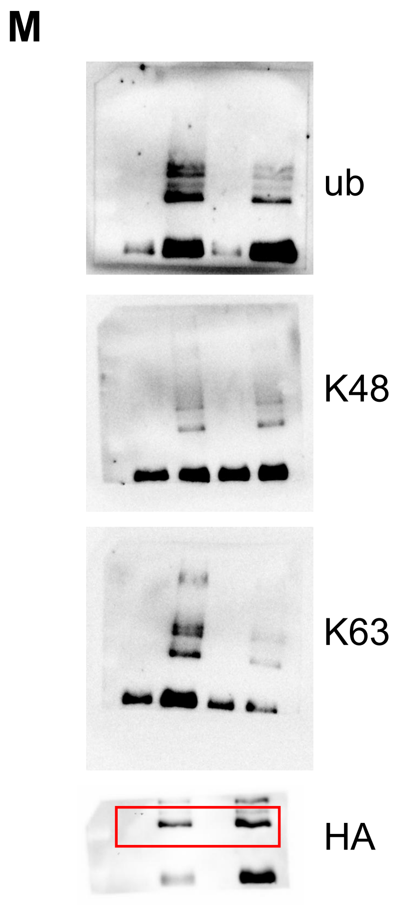

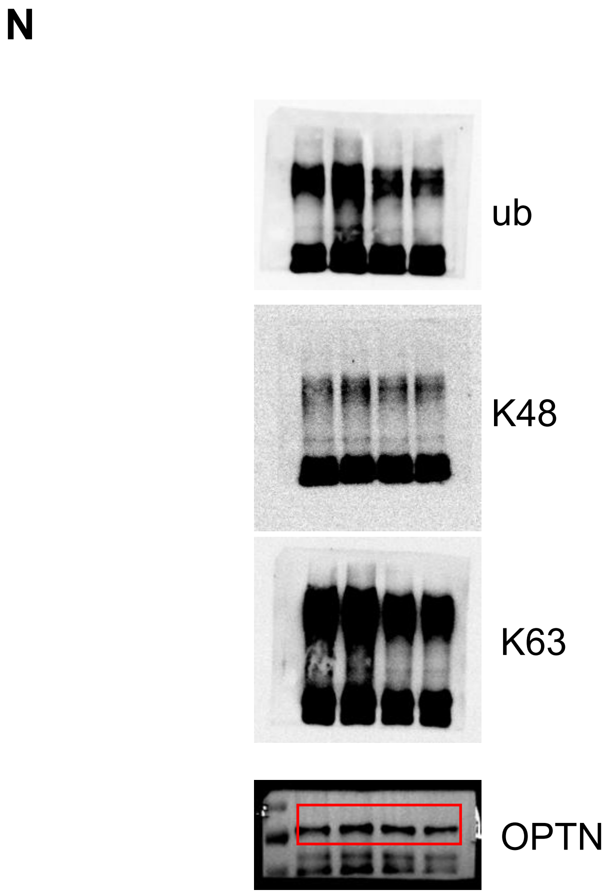

Fig. 8 RNF8 regulated the degradation of KDM6A through ubiquitination OPTN.

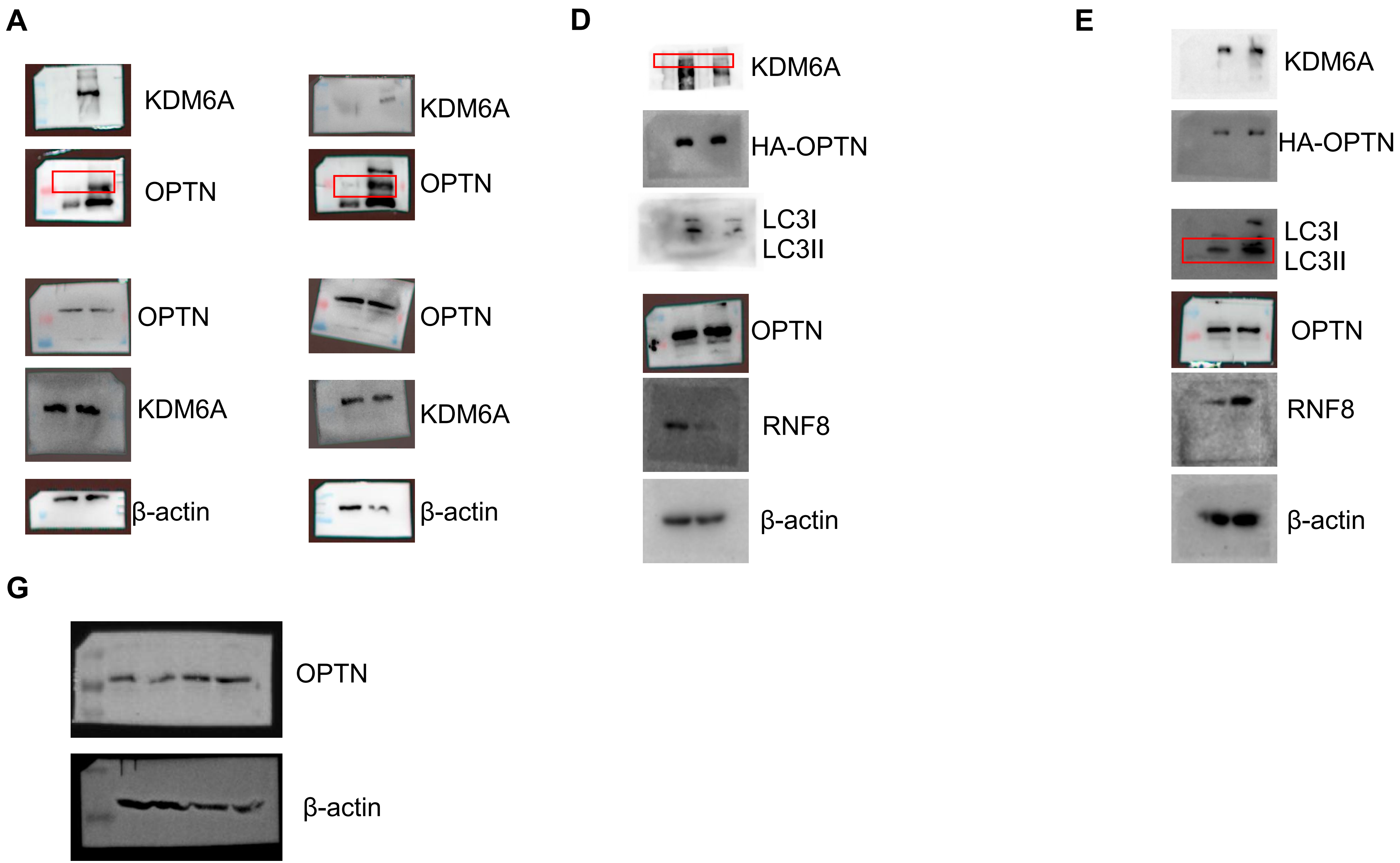

Fig. 8 RNF8 regulated the degradation of KDM6A through ubiquitination OPTN.

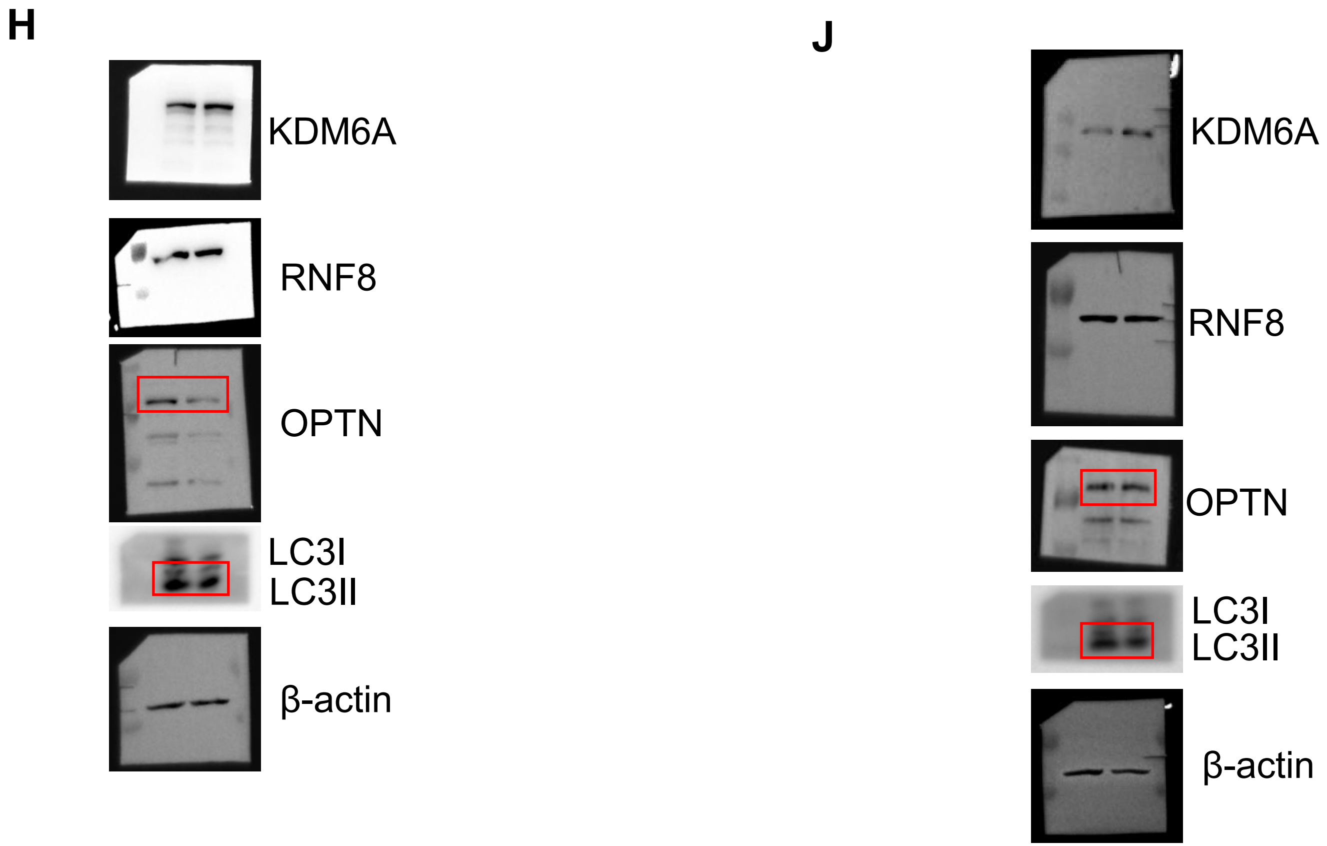

Sup.3 LPS-induced systemic immune response impaired differentiation of testicular spermatogenic cells.

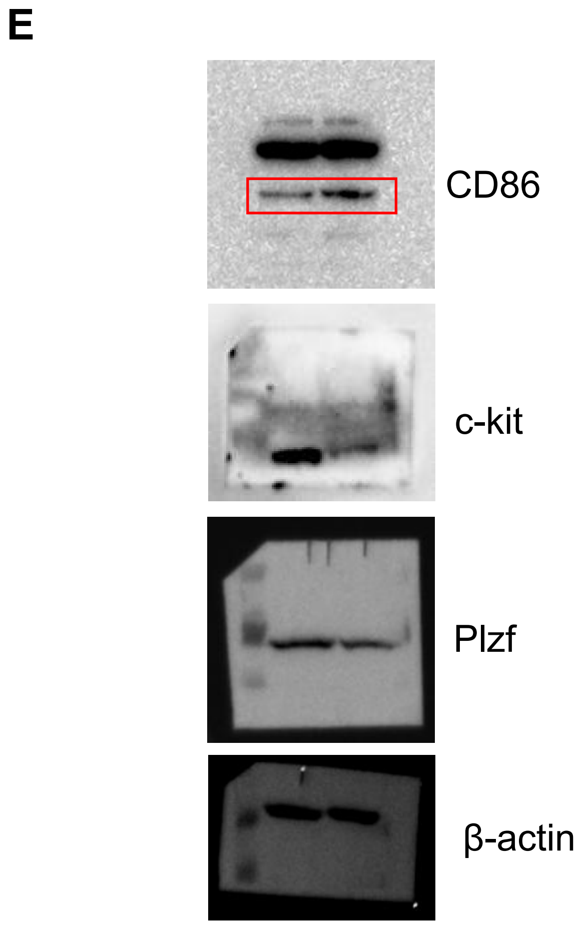

Sup. 4 Macrophage pro-inflammatory polarization led to enhanced inflammation in testicular microenvironment and increased damage in spermatogenic cells.

C

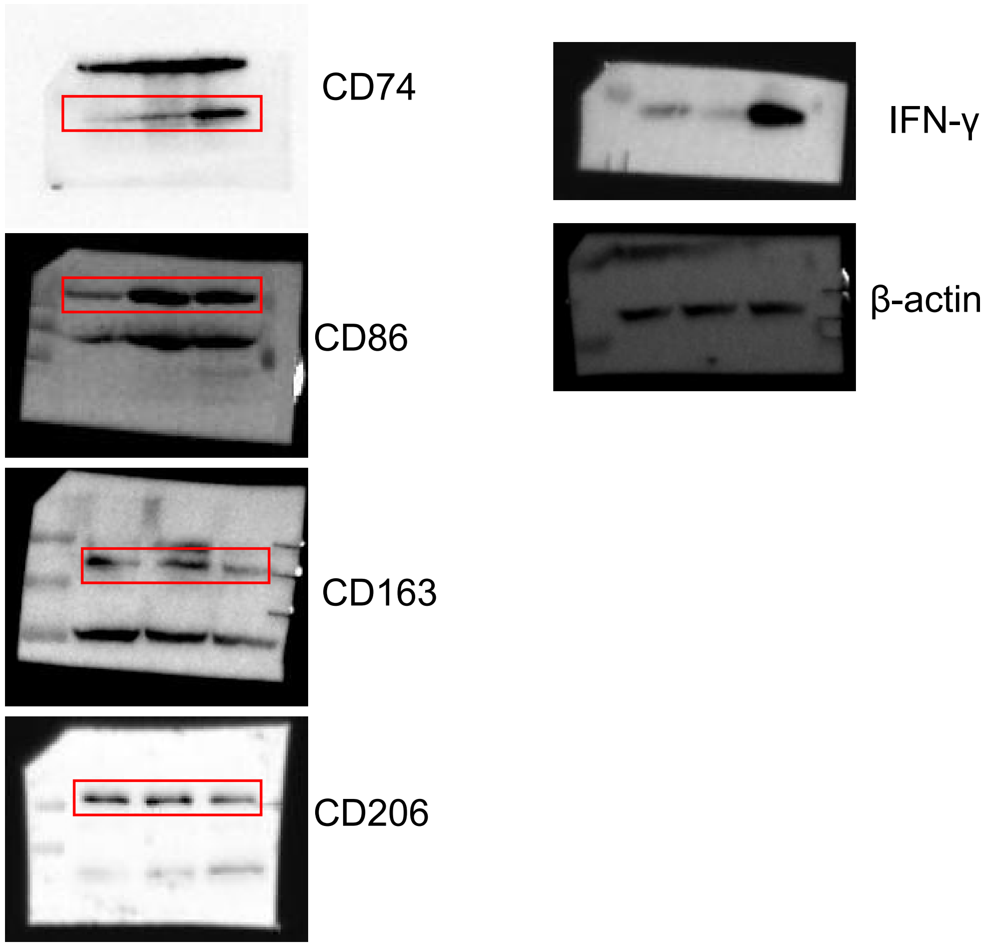

G

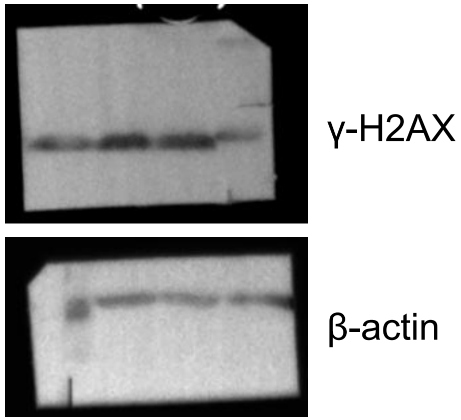

Sup. 5 Proinflammatory polarization of macrophages led to exhaustion in the differentiation of testicular spermatogenic cells.

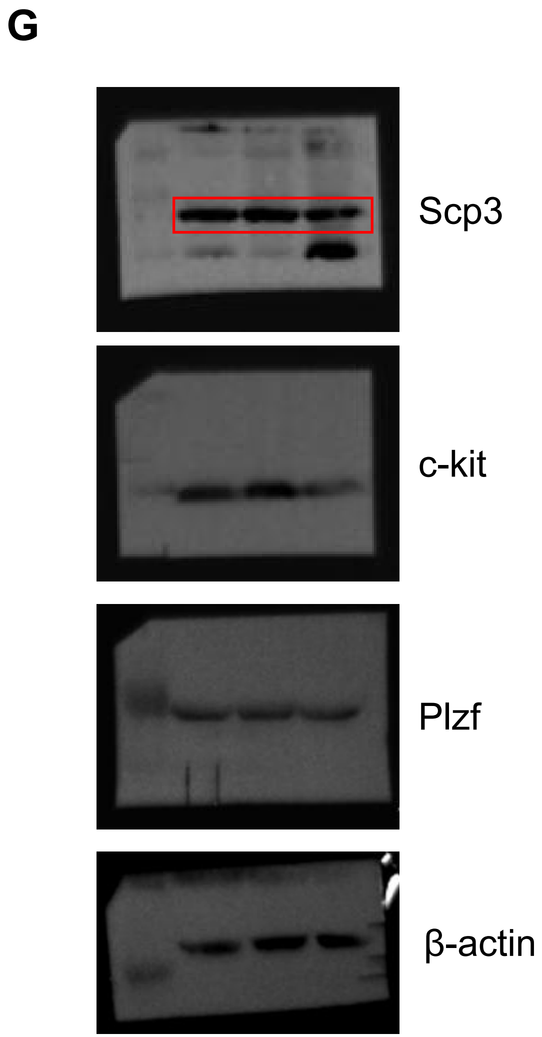

Sup. 7 RNF8 affected macrophage pro-inflammatory polarization by regulating KDM6A.

B

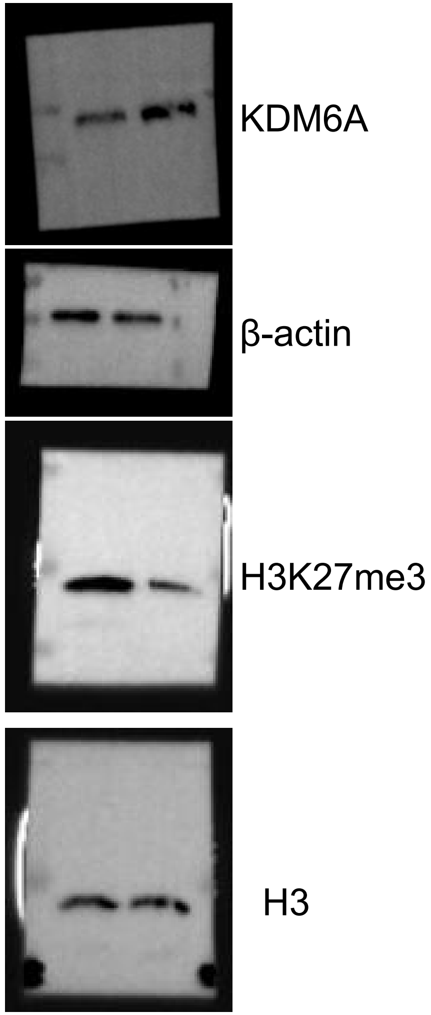

D

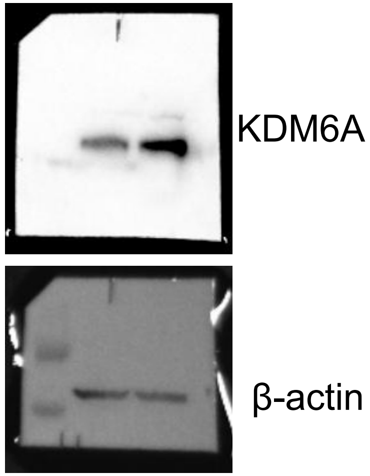

Sup. 9 The expression of RNF8 in cytoplasm promoted autophagy progression.

A

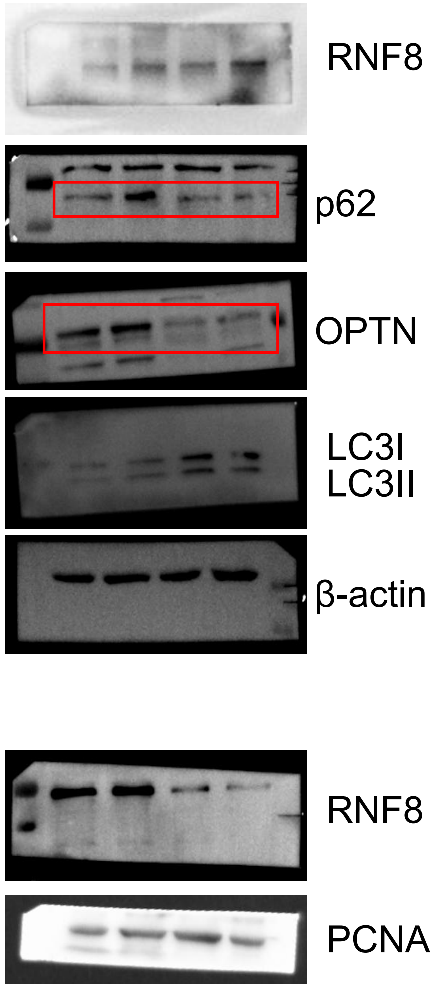

C

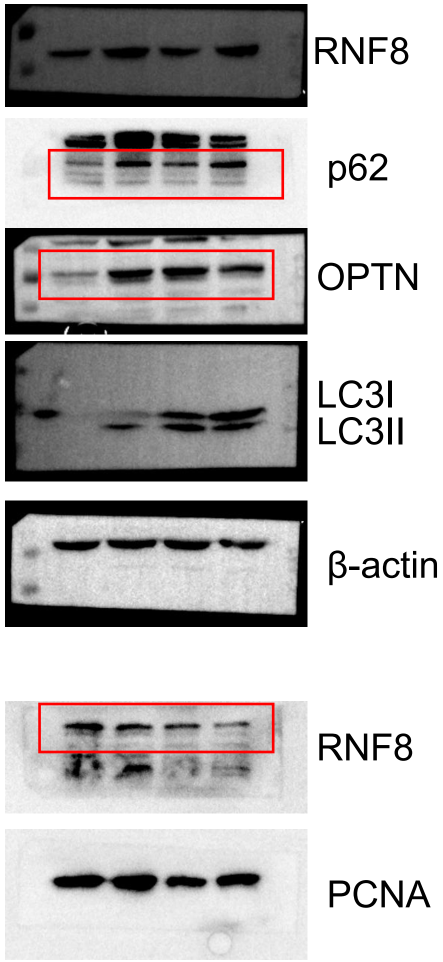

Sup. 9 The expression of RNF8 in cytoplasm promoted autophagy progression.

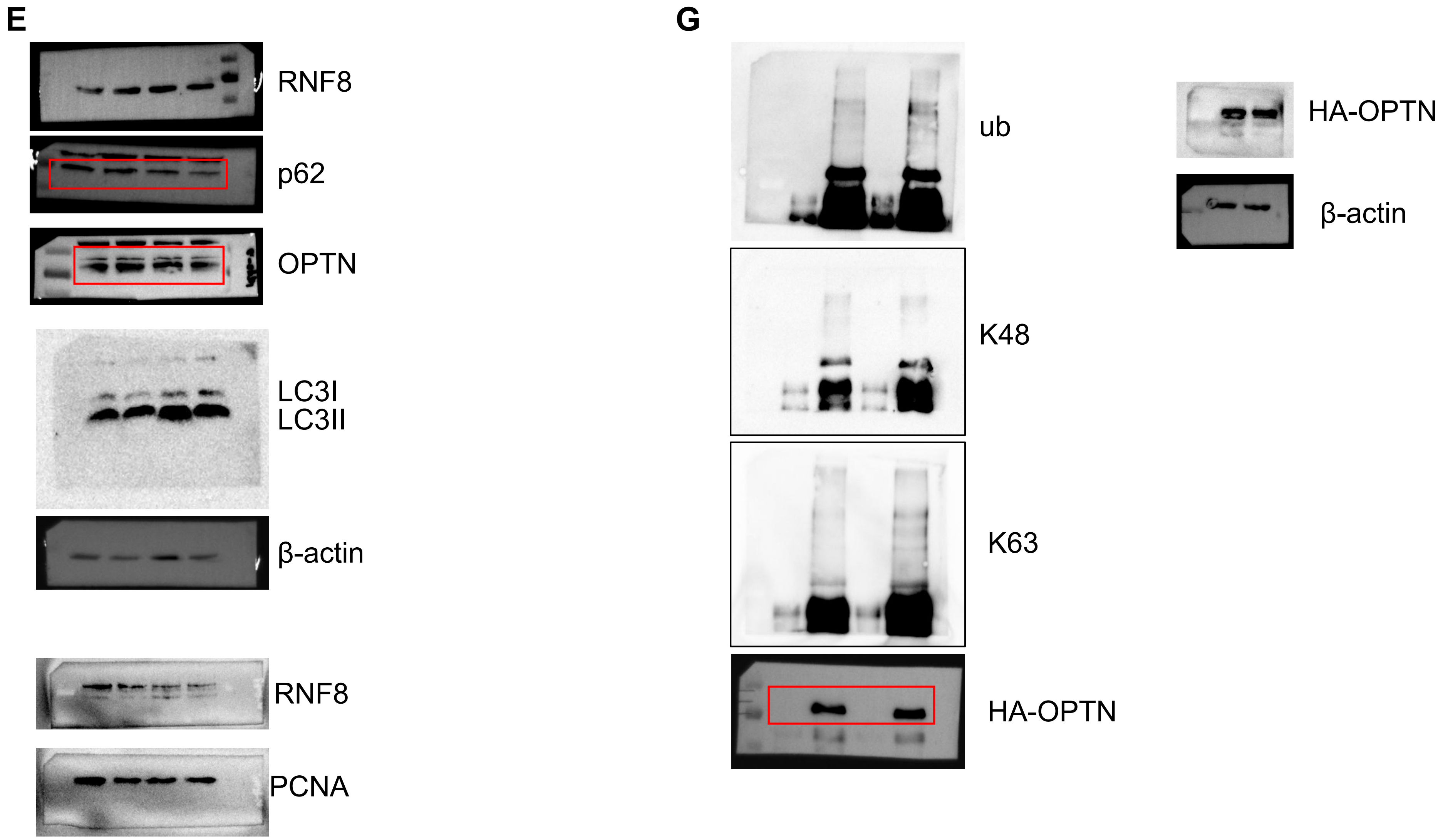

Supplement: Supplementary file 2 — Original Data [file 41420_2025_2641_MOESM2_ESM.pdf]
